# Synthesis and Evaluation of Antitumor and Anti-Angiogenesis Activity of Pyrone- or Pyridone-Embedded Analogs of Cortistatin A

**DOI:** 10.3390/md23040179

**Published:** 2025-04-20

**Authors:** Yuri Fujimoto, Kanako Mizuno, Yuta Nakamura, Masayoshi Arai, Naoyuki Kotoku

**Affiliations:** 1 College of Pharmaceutical Sciences, Ritsumeikan University, 1-1-1 Noji-higashi, Kusatsu 525-8577, Shiga, Japan; ph0162fp@ed.ritsumei.ac.jp (Y.F.); ph0187si@ed.ritsumei.ac.jp (Y.N.); 2 Graduate School of Pharmaceutical Sciences, Osaka University, 1-6 Yamadaoka, Suita 565-0871, Osaka, Japan; m.kombbbbb@gmail.com (K.M.); araim@phs.osaka-u.ac.jp (M.A.)

**Keywords:** Cortistatin A, simplified analog, antitumor effect, anti-angiogenic substance

## Abstract

Simplified analogs of cortistatin A were synthesized and biologically evaluated to develop novel antitumor substances that target angiogenesis. To analyze the effect of substituents at positions corresponding to C-2 and/or C-4 of the A-ring, various pyrone- or pyridone-embedded analogs were designed and synthesized. Among the prepared analogs, the pyridone analog **19** bearing a methyl group at C-2 and a hydroxyl group at C-4 showed potent and selective growth inhibitory activity against human umbilical vein endothelial cells (HUVECs, IC_50_ = 0.001 µM, selective index over that against human epidermoid carcinoma KB3-1 cells = 6400), exceeding those of natural products. The analog **19** of oral administration exhibited excellent in vivo antitumor activity in mice subcutaneously inoculated with sarcoma S180 cells.

## 1. Introduction

Marine natural products have garnered considerable attention as rich and promising sources of drug candidates, particularly in the field of anticancer drug discovery [1,2]. Generally, novel bioactive compounds are identified through bioassay-guided separation using simple phenotype screening. Phenotypic screening is a promising method for the discovery of first-in-class drug candidates with a novel mode of action, and the mechanistic study of these compounds might reveal an unknown signaling pathway as a target for drug discovery [3].

However, only small amounts of bioactive compounds can be isolated from the extracts of marine organisms such as sponges and tunicates, and the sustainable supply of active compounds has been a challenge for further evaluation and drug development. Chemical synthesis of bioactive natural products and their analogs can overcome this drawback. In contrast, natural products often exhibit diverse bioactivities by binding to multiple target molecules (proteins) because of their complex chemical structures containing various functional groups. Successful examples indicate that the truncation of its partial structure or several moieties is effective, in some cases, for extracting the essential scaffold of the natural product, resulting in a reduction in the number of target proteins without losing specific bioactivity. In addition, structural simplification leads to the supply of a substantial amount of the compound for further studies such as in vivo activity evaluation and mechanistic analysis. Furthermore, the Absorption, Distribution, Metabolism, Excretion, and Toxicity (ADMET) profiles of the compounds can be improved by reducing the molecular weight [4,5,6]. Therefore, the syntheses of truncated natural products based on structure–activity relationship (SAR) studies are expected to develop more accessible and promising drug leads with optimized activity or chemical stability.

**Figure 1:**
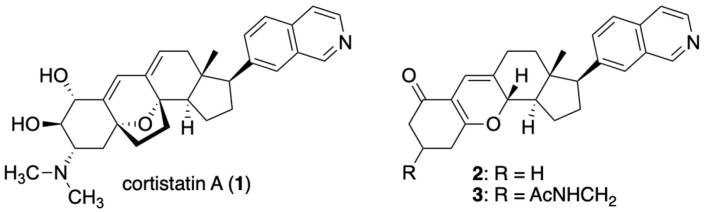
The chemical structure of cortistatin A (**1**) and its simplified analogs (**2,3**).

Angiogenesis is a critical phenomenon in tumor growth and metastasis. A growing tumor must form new blood capillaries that provide nutrients and oxygen. New blood vessels are also used as a way for tumor cells to enter the circulation, resulting in cancer metastasis to other organs. Therefore, substances that inhibit angiogenesis have considerable potential as novel therapeutic agents for cancer treatment [7]. Previously, our research group focused on identifying anti-angiogenic substances as novel anti-cancer drug seeds from marine organisms and isolated cortistatins, a family of novel *abeo*-9(10-19)-androstane-type steroidal alkaloids, from the Indonesian marine sponge *Corticium simplex* [8,9,10]. Cortistatin A (**1**, Figure 1), a major constituent of *C. simplex*, shows potent and highly selective antiproliferative activity against human umbilical vein endothelial cells (HUVECs). Compound **1** also exhibited potent in vitro anti-angiogenic activity and inhibited the migration and tubular formation of HUVECs induced by vascular endothelial growth factor (VEGF) or basic fibroblast growth factor (bFGF). In addition, mechanistic study implied that the selective growth inhibitory activity of **1** against HUVECs is independent on the VEGF signal pathway, one of the main pathways of angiogenesis and thus the target of most existing anti-angiogenesis drugs [11].

Because of their unique chemical and biological properties, cortistatins are promising novel anti-angiogenic drug leads. However, the supply of cortistatins from natural sources to validate the feasibility of their use as potential drug leads is impractical. Although many synthetic studies on cortistatins [12,13,14] including six total syntheses [15,16,17,18,19,20] have been reported, the overall yields were insufficient in most cases. Therefore, we focused on developing structurally simplified analogs and identified a useful compound, **2** (Figure 1). Analog **2** exhibited good antiproliferative activity in HUVECs with significant selectivity and potent in vivo anti-tumor activity following oral administration [21]. Since then, we have focused on developing a more promising anticancer drug lead through structural optimization of analog **2** as a scaffold and establishing a more practical synthetic method [22]. This resulted in the generation of a novel analog with potent activity and selectivity exceeding those of natural cortistatin A (**1**), as well as significant in vivo anti-angiogenic activity, the detailed results of which are presented in this report.

## 2. Results and Discussions

### 2.1. Design of Pyrone- or Pyridone-Embedded Analogs

**Figure 2:**
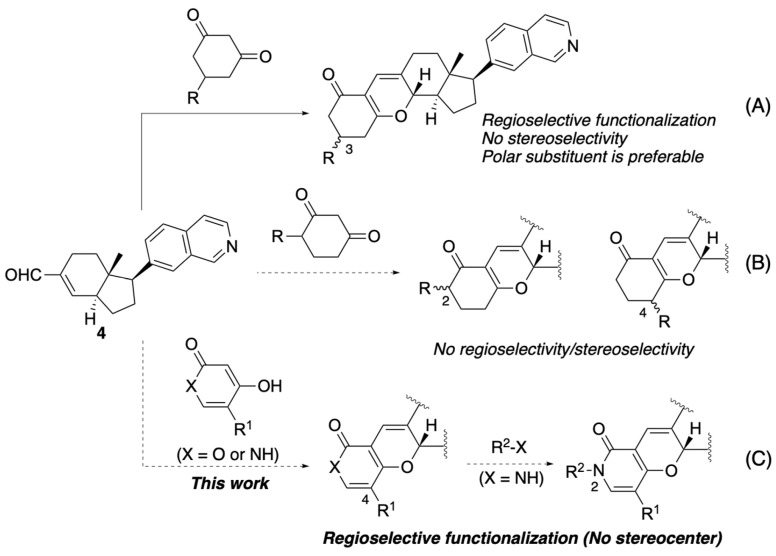
(**A**) General synthetic strategy of cortistatin A analog with a substituent at C-3. (**B**) Synthetic strategy of analogs with C-2 or C-4 substituent. (**C**) Synthetic strategy of novel analogs with substituents at C-2 or C-4 in this work using 4-hydroxy-2-pyrone or pyridone.

As shown in Figure 1, the anthracene-like planar ABC-ring system of simplified analogs **2** and **3** developed by our group mimics the core structure of **1**, an *abeo*-9(10-19)-androstane-type rearranged steroidal backbone with an oxa-bridge between the 5- and 8-position. The tricyclic structure was prepared by Knoevenagel condensation of aldehyde **4** and cyclohexane-1,3-dione derivatives (Figure 2A). Considering the σ-symmetry of cyclohexane-1,3-dione, regioselective functionalization of the A-ring was possible only at the C-3 position of the natural product using 5-substituted cyclohexane-1,3-diones. As the analog **3**, with a acetamide moiety at C-3, exhibited potent growth inhibitory activity against HUVECs (IC_50_ = 0.0026 µM) and high selectivity over KB3-1 cells (IC_50_ = 8.2 µM, selective index (S.I.) = 3150), synthesis and evaluation of the analogs with C-2 and/or C-4-substitutents are highly desirable.

However, the above strategy might not be effective for preparing C-2- or C-4-substituted analogs because the Knoevenagel condensation between aldehyde **4** and 4-substituted cyclohexane-1,3-dione derivatives provides a mixture of two regioisomers in an uncontrolled manner (Figure 2B). Furthermore, no stereocontrol of the newly formed asymmetric center was achieved in either case.

Subsequently, we designed novel type of analogs in this work, as shown in Figure 2C. 4-Hydroxy-2-pyrone possesses a cyclic β-keto ester structure that may function as a surrogate for the cyclohexane-1,3-dione derivative in the synthesis of the analogs. The asymmetric nature of the 4-hydroxy-2-pyrone system gives the desired cyclized product with a unified structure, and the substituent at C-5 of the pyrone (R^1^ in Figure 2C) leads to 2-oxa analogs with a substituent at C-4. The resulting AB-ring system, the 2*H*,5*H*-pyrano[4,3-*b*]pyran-5-one structure, has been observed in some meroterpenoid natural products, and successful total synthesis of the compounds was achieved through the formal [3 + 3] cycloaddition method [23,24].

Another promising alternative to the A-ring unit is 4-hydroxy-2-pyridone with a cyclic β-keto amide structure, and condensation-electrocyclization with aldehyde **4** would afford aza-analogs. Similarly, the synthesis of 2,6-dihydro-5*H*-pyrano[3,2-*c*]pyridin-5-one derivatives leads to natural products or bioactive compounds have been reported [25,26,27,28,29]. The nitrogen atom of pyridone corresponds to C-2 of the natural product and can be used as a base for another substituent R^2^ to analyze the effect of verification at this position (Figure 2C).

### 2.2. Synthesis of Pyrone- or Pyridone-Embedded Analogs of Cortistatin A

#### 2.2.1. Synthesis of Pyrone-Embedded Analogs

**Scheme 1:**
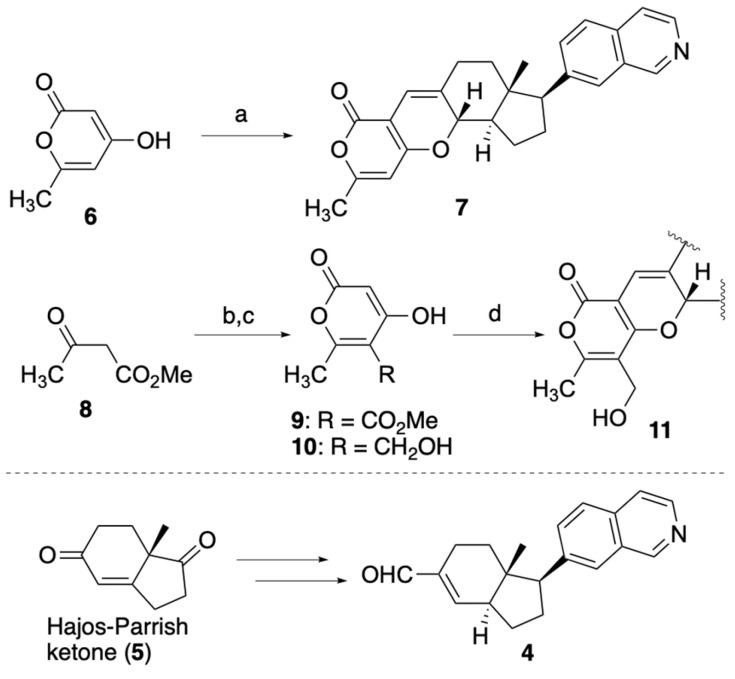
Synthesis of pyrone-embedded analogs **7** and **11**. Reagents and conditions: (a) **4**, piperidine, 1,4-dioxane, 54%; (b) malonyl chloride, CH_2_Cl_2_, 74%; (c) BH_3_·S(CH_3_)_2_, CH_2_Cl_2_/THF, 78%; (d) **4**, piperidine, 1,4-dioxane, 76%.

Based on the above strategy, the synthesis of pyrone-embedded analogs was performed first, as shown in Scheme 1. Aldehyde **4**, the common precursor of all the analogs in this work, was prepared from (+)-Hajos-Parrish ketone (**5**) through a procedure developed by our group [22]. Commercially available 4-hydroxy-6-methyl-2*H*-pyran-2-one (**6**) was reacted with **4** to afford the desired pyrone-embedded analog **7** in moderate yield. The use of piperidine as a base gave a better reaction yield than ethylenediamine, which was the optimized base in our previous work using cyclohexane-1,3-dione derivatives as A-ring parts [21]. Another analog, **11**, with a hydroxymethyl moiety at C-4, was obtained in a similar manner using the corresponding pyrone derivative **10**. According to literature [30], ethyl acetoacetate (**8**) reacts with malonyl chloride to give compound **9** in good yield. Subsequent reduction of the ester moiety of **9** with BH_3_ [31] and condensation cyclization of the resulting compound **10** with aldehyde **4** afforded the analog **11** in good yield.

#### 2.2.2. Synthesis of Pyridone-Embedded Analogs

**Scheme 2:**
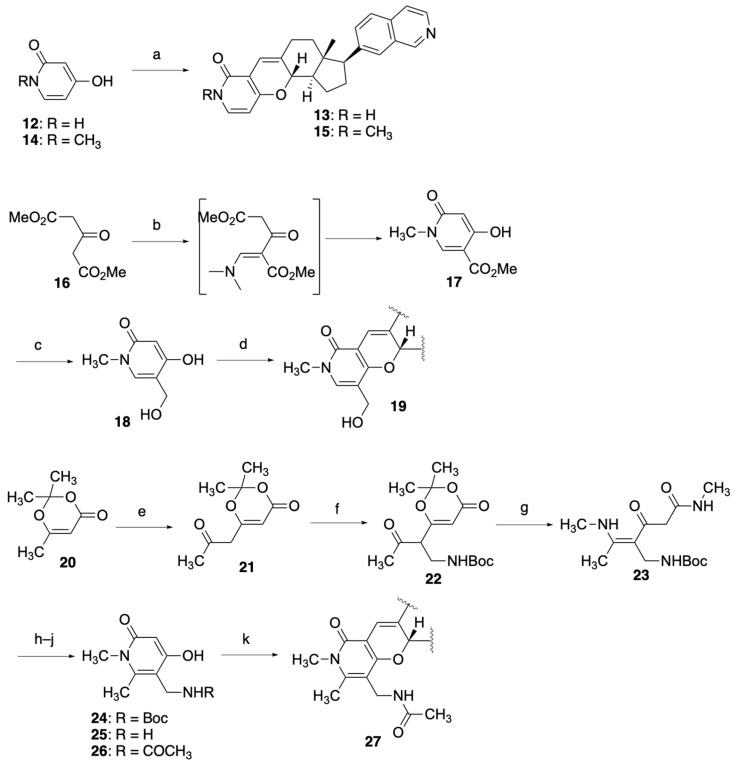
Synthesis of pyridone-embedded analogs. Reagents and conditions: (a) ethylenediamine diacetate, DMF, 27% for **13**; 57% for **15**; (b) *N*,*N*-dimethylformamide dimethyl acetal, CH_3_NH_2_, MeOH, 37%; (c) DIBAL, toluene, −78 °C, 39%; (d) ethylenediamine diacetate, DMF, 45%; (e) LHMDS, AcCl, THF, 50%; (f) *N*-Boc(tosyl)methylamine, NaH, THF, 55%; (g) CH_3_NH_2_, toluene, 75 °C, 50%; (h) CH_3_CO_2_H, toluene; (i) TFA, CH_2_Cl_2_; (j) Ac_2_O, K_2_CO_3_, DMF, 34% (3 steps); (k) ethylenediamine diacetate, DMF, 30%.

Next, the synthesis of the pyridone-embedded analogs was attempted. Analog **13**, which has the simplest A-ring structure, was obtained by condensation of commercially available pyridine-2,4-diol (**12**) and aldehyde **4** (Scheme 2). Ethylenediamine diacetate was effective instead of piperidine in this case, although the reaction yield was not very high. *N*-Methyl analog **15** was obtained in the same manner using 4-hydroxy-1-methylpyridin-2(1*H*)-one (**14**), which was prepared from **12** [32].

**Scheme 3:**
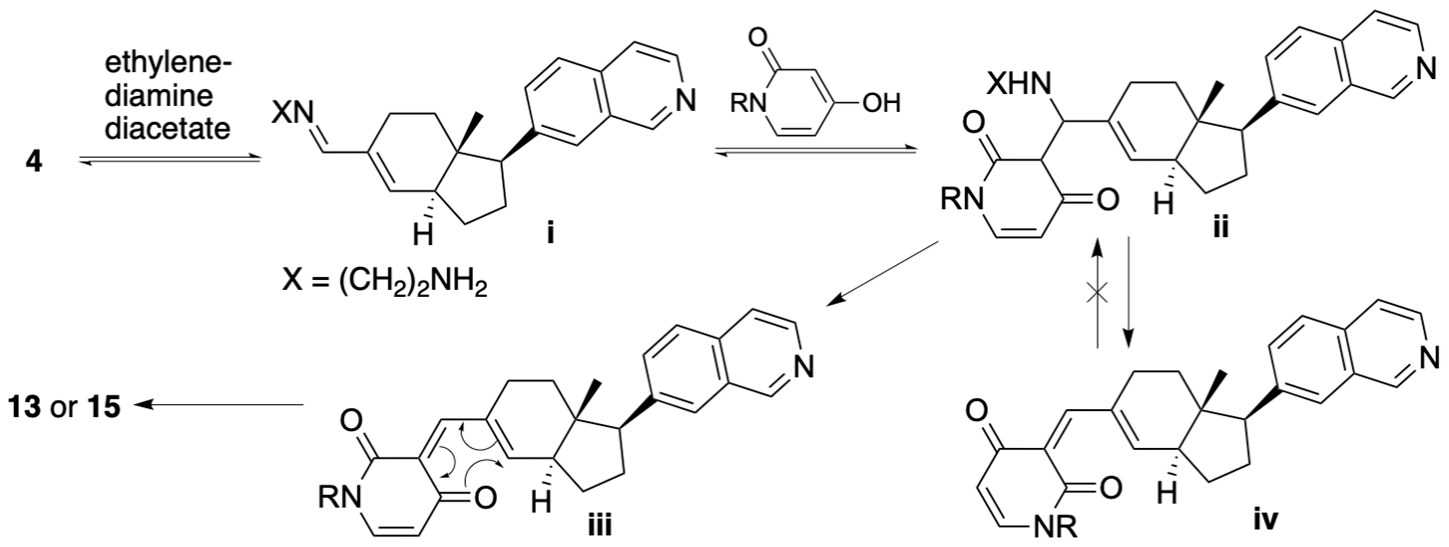
Knoevenagel condensation-electrocyclization of aldehyde **4** and pyridone derivatives.

Tracking the condensation reaction revealed that compound **iv**, the geometrical isomer of the desired Knoevenagel condensation product **iii** giving the electrocyclization product, was the main byproduct (Scheme 3). This means that the reaction is not in equilibrium, and the yield of the analog depends on the ratio of **iii** and **iv**. Indeed, no reaction was observed after the ethylenediamine treatment of isolated compound **iv**.

The pyridone-embedded analogs with polar substituents at C-4 were synthesized as follows: According to the literature [33], dimethyl acetone-1,3-dicarboxylate (**16**) was reacted with *N*,*N*-dimethylformamide dimethyl acetal to provide an enaminoketone, and subsequent cyclization occurred to give compound **17** by the treatment with methylamine. The ester moiety of **17** was then converted into a primary hydroxyl group via DIBAL reduction, yielding compound **18**. Finally, condensation with aldehyde **4** afforded the desired analog **19**.

Furthermore, analog **27** with an acetamide moiety at C-4 was prepared through a separate reaction because the substitution of the hydroxyl group of **19** with a nitrogen-based functionality under various conditions did not give satisfactory results. The acetylation of commercially available 2,2,6-trimethyl-4*H*-1,3-dioxine-4-one (**20**) afforded compound **21** [34]. The dienolate ion derived from **21** was then reacted with *N*-Boc(tosyl)methylamine to give compound **22** with a nitrogen-containing side chain in moderate yield [35]. Subsequent treatment with an excess amount of methylamine provided enamine-amide **23**, which was annulated in the presence of acetic acid by heating to give pyridone derivative **24**. The one-pot transformation of **22** to **24**, methylamine treatment, and subsequent heating did not yield satisfactory results. Removal of the Boc group of **24** using trifluoroacetic acid (TFA) and subsequent treatment of amine **25** with acetic anhydride under basic conditions provided compound **26**, the target A-ring fragment with an acetamidomethyl moiety at the C-4 position. Finally, condensation cyclization with aldehyde **4**, catalyzed by ethylenediamine diacetate, afforded desired analog **27**.

### 2.3. Biological Evaluation of Pyrone- or Pyridone-Embedded Analogs

#### 2.3.1. Antiproliferative Activities of the Analogs Against Endothelial or Cancer Cells

The antiproliferative activities of the synthetic analogs against HUVECs and human epidermoid carcinoma KB3-1 cells were evaluated and the results are summarized in Table 1. It revealed that pyrone-embedded analogs **7** and **11** exhibited moderate or good antiproliferative activity against HUVEC (IC_50_ = 0.09 and 0.02 μM, respectively). Similar to the C-3 substitution described in our previous study (**2** vs. **3**) [22], the polar substituent introduced at C-4 was found to have a positive effect. In contrast, analog **7** showed weakened cytotoxicity against KB3-1 cells (IC_50_ = 62.8 μM). Compound **13** showed 9-fold higher potency than **7**, indicating that pyridone might be a more suitable A-ring structure than pyrone. The additional 3.3-fold potency of analog **15** over **13** clearly shows that *N*-methylation has a positive effect. Surprisingly, the introduction of a nonpolar substituent at that position increased bioactivity because cortistatin A (**1**) has a hydroxyl group at C-2. The introduction of a polar substituent at C-4 further enhanced the antiproliferative activity against HUVECs (analog **19** over **15**), the IC_50_ value of which exceeded that of the natural product without losing cell selectivity (6400 selective index value (IC_50_ against KB3-1 cells/IC_50_ against HUVECs)). Analog **27**, with an acetamide moiety at C-4, showed weaker activity against HUVECs (IC_50_ = 0.01 μM), whereas the same substituent at C-3 exhibited greater enhancing effect in the case of the analog **3** [22].

In 2015, Shair et al. reported that cortistatin A (**1**) exhibited potent antiproliferative activity against some types of acute myeloid leukemia (AML) cells through the inhibition of cyclin-dependent kinase 8 (CDK8). They also showed that most solid tumor cell lines were not affected by treatment with **1**, although the effect of the compound on endothelial cells was not tested [36]. Subsequently, simplified steroidal analogs of **1** were found to exhibit similar potent inhibitory activity against CDK8 by Gray et al. in 2018. They also developed a proteolysis-targeting chimera (PROTAC) derived from a steroidal analog as an effective CDK8 [37].

**Figure 3:**
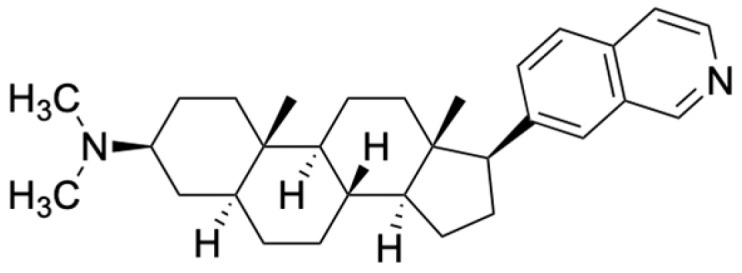
The chemical structure of a steroidal analog (**28**).

To analyze the role of CDK8 in the proliferation of HUVECs, we prepared compound **28** (Figure 3), a steroidal analog, as a potent inhibitor of CDK8 (IC_50_ = 24 nM) [37] and evaluated its antiproliferative activities against HUVECs and KB3-1 cells. The analog **28** showed weak antiproliferative activity against HUVECs (IC_50_ = 0.56 µM) and low selectivity over KB3-1 cells (Table 1). This result implies that CDK8 contributes less to the proliferation of HUVECs at this time, although it is unclear whether the difference in activity between CDK8 inhibition and HUVECs growth inhibition is caused by the presence of another molecule responsible for the proliferation of HUVECs or by the low cell membrane permeability of **28**.

#### 2.3.2. In Vivo Activity Evaluation of Analog **19**

**Figure 4:**
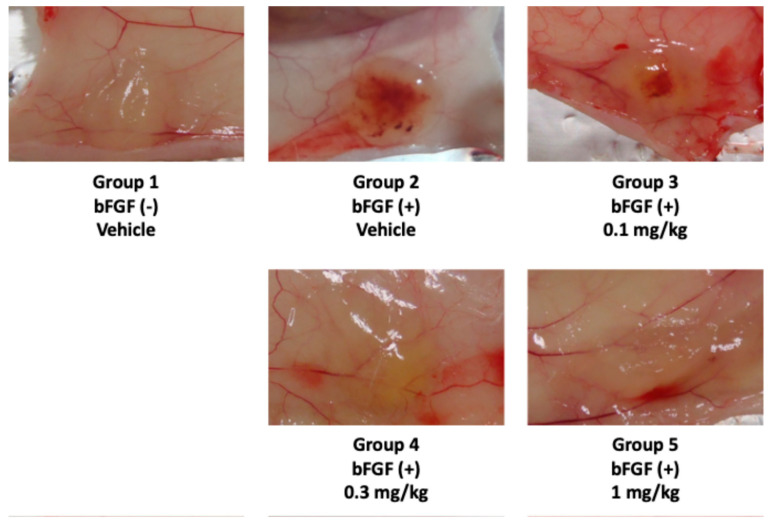
Pictures of the representative implanted Matrigel of the control and the treated group with the indicated amount of analog **19**.

We evaluated in vivo activity of compound **19**, the most potent analog tested in this study, on the formation of new blood vessels, by Matrigel plug assay [21] in mice. As shown in Figure 4, oral administration of compound **19** prevented the formation of new blood capillaries in the Matrigel plug induced by bFGF. Compound **19** significantly decreased the hemoglobin content in the plug even after administration of 0.1 mg/kg administration, and the 1 mg/kg dose of **19** reduced the hemoglobin content to the same level as the negative control. These results indicate that analog **19** effectively inhibited in vivo angiogenesis.

During the assay, the mean body weights of mice in both the control and compound-treated groups were measured to determine the acute toxicity of the analogs. As shown in Table 2, no body weight loss was observed in any group even after day 9 of the treated group of 1 mg/kg administration. In addition, the hepatic toxicity of the compound was evaluated by measuring the activities of aspartate aminotransferase (AST) and alanine aminotransferase (ALT) in mouse serum on day 9. No significant changes in those enzyme activities were observed between the 1 mg/kg treatment group (AST: 57.8 ± 4.5 IU/L, ALT: 48.4 ± 3.3 IU/L) and control group (AST: 59.6 ± 3.1 IU/L, ALT: 43.6 ± 4.7 IU/L). These results indicated that analog **19** exhibited potent antiangiogenic activity in vivo without severe toxicity.

**Figure 5:**
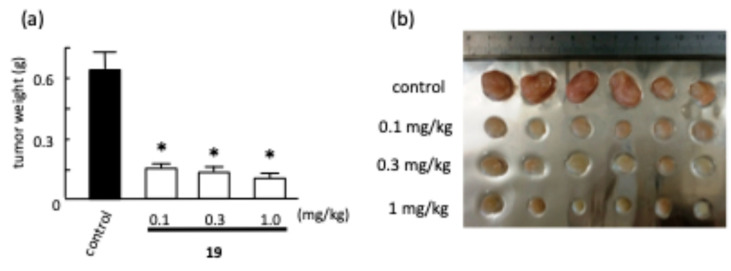
In vivo anti-tumor effect of analog **19**. (**a**) Tumor weight of each group (mean ± SD); *: *p* < 0.01. (**b**) Image of surged tumors after two weeks.

We further examined the in vivo antitumor effects of analog **19**. Orally administered compound **19** of only 0.1 mg/kg exhibited significant tumor growth inhibition, with ~75% reduction compared to the control group, and more than 85% tumor weight reduction was observed with 1 mg/kg administration (Figure 5). Similar to the Matrigel plug assay, body weight loss and diarrhea, which are typical signs of acute toxicity, were not observed. The results of these in vivo experiments indicated that the potent anti-tumor activity of analog **19** was caused by the strong inhibition of angiogenesis promoted by the implanted tumor.

## 3. Materials and Methods

### 3.1. General

The following instruments were used to obtain physical data: a JASCO (Tokyo, Japan) DIP-370 digital polarimeter (L = 50 mm) for specific rotations; a JEOL (Tokyo, Japan) JNM-ECZ500R/S1 (^1^H-NMR: 500 MHz, ^13^C-NMR: 125 MHz) spectrometer or an Agilent (Santa Clara, CA, USA) NMR system (^1^H-NMR: 600 MHz, ^13^C-NMR: 151 MHz) for ^1^H and ^13^C NMR data, using tetramethylsilane or solvent residual peaks (CDCl_3_: 7.26/77.0 ppm; DMSO-*d*_6_: 2.49/39.5 ppm; CD_3_OD: 3.31/49.0 ppm) as internal standards; Waters (Milford, MA, USA) Q-Tof Ultima API or Xevo G2-XS Q-Tof mass spectrometer for ESI-TOF MS. Silica gel 60N (Kanto (Tokyo, Japan) 63–210 μm) and pre-coated thin layer chromatography (TLC) plates (Merck (Darmstadt, Germany) 60F_254_) were used for column chromatography and TLC, respectively. Spots on the TLC plates were detected by spraying with an acidic *p*-anisaldehyde solution (*p*-anisaldehyde: 25 mL, *c*-H_2_SO_4_: 25 mL, AcOH: 5 mL, EtOH: 425 mL) or with a phosphomolybdic acid solution (phosphomolybdic acid: 25 g, EtOH: 500 mL) with subsequent heating. Compounds **4** [22], **9** [30], **10** [31], **14** [32], **17** [33], and **21** [34] were prepared according to the literature. Other chemicals were purchased from Sigma-Aldrich (St. Louis, MO, USA), Nacalai Tesque, Inc. (Kyoto, Japan), Tokyo Chemical Industry Co., Ltd. (Tokyo, Japan), or FUJIFILM Wako Pure Chemical Co. (Osaka, Japan). Hard copies of ^1^H and ^13^C NMR spectra can be found as Supplementary Materials.

### 3.2. Assay for Cytotoxicity of Compounds Against HUVECs

HUVECs (5 × 10^5^ cells/vial) were purchased from Kurabo Inc. and grown in HuMedia-EG2 medium with growth supplements (Kurabo, Osaka, Japan). HUVECs in the culture medium were plated into each well of 96-well plates (2 × 10^3^ cells/well/100 µL). After 24 h, the serially diluted compounds as a 5% DMSO/EtOH or EtOH solution (1 µL) were added, and then the plates were incubated for an additional 72 h in a humidified atmosphere of 5% CO_2_ at 37 °C. The cell proliferation was detected by WST-8 colorimetric reagent (Nacalai Tesque, Inc., Kyoto, Japan). The IC_50_ value was determined by linear interpolation from the growth inhibition curve, and the averaged IC_50_ values of the triplicate experiment are shown in Table 1.

### 3.3. Antiproliferative Activity of the Compounds Against Cancer Cells

KB3-1 cells were cultured in RPMI-1640 medium supplemented with 10% heat-inactivated fetal bovine serum and kanamycin (50 μg/mL). Cells were plated into 96-well microplates at 2 × 10^3^ cells/100 μL assay medium/well, and various concentrations of test compounds were added to each well as a 5% DMSO/EtOH or EtOH solution (1 µL). The plates were incubated at 37 °C in a humidified atmosphere of 5% CO_2_ for 72 h, and cell proliferation was determined by WST-8 colorimetric assay as above, and the averaged IC_50_ values of the triplicate experiment are shown in Table 1.

### 3.4. In Vivo Matrigel Plug Assay and Toxicity Tests of Analog **19**

All animal procedures were approved by the Committee on Animal Experimentation of Osaka University (Approval number: DouYaku 24-8-1). The measurements of in vivo angiogenesis using Matrigel plugs were performed as described previously [21]. An aliquot (250 µL) of Matrigel containing 200 ng/mL of bFGF and 100 U/mL of heparin was injected subcutaneously into the ventral side of female ddY mice (6 weeks old, *N* = 4, Japan SLC, Inc., Shizuoka, Japan). Then, analog **19** was orally administered on every other day (total 5 times: day 1, 3, 5, 7, and 9) as a suspension in 1% sodium carboxymethyl cellulose (CMC-Na). At day 10, the Matrigel plugs were photographed and removed. The levels of new vessels were quantified by measuring the hemoglobin of the Matrigel plug (µg hemoglobin/mg Matrigel) with the Drabkin reagent Kit.

The body weight of mice of the control group (bFGF (+) or (−)) and the groups treated with the testing compound (0.1, 0.3, and 1 mg/kg) at day 1 and day 9 were measured. The mean activities of aspartate aminotransferase (AST) and alanine aminotransferase (ALT) in mouse serum at day 10 of control group and the 1 mg/kg treated group were measured using the LabAssay^TM^ AST or ALT kit (FUJIFILM Wako Pure Chemical Co., Osaka, Japan), respectively.

### 3.5. In Vivo Antitumor Effect of Analog **19**

Murine sarcoma S180 cells (1 × 10^6^ cells/body) were implanted subcutaneously into the right ventral flank of female ddY mice (6 weeks old). After one week from implantation, analog **19** was orally administered on every other day for 14 days (total 7 times) as a suspension in 1% CMC-Na. Then, the tumor was isolated and weighed to calculate the inhibition ratio. The control group and the group treated with the testing compound consisted of six mice each in this study.

### 3.6. Synthesis

#### 3.6.1. (3*S*,3a*R*,11a*S*,11b*R*)-3-(Isoquinolin-7-yl)-3a,9-dimethyl-1,2,3,3a,4,5,11a,11b-octahydro-7*H*-cyclopenta[h]pyrano[4,3-b]chromen-7-one (**7**)

4-Hydroxy-6-methyl-2-pyrone (**6**, 25.0 mg, 0.20 mmol) and piperidine (9.8 µL, 0.10 mmol) were added to a solution of **4** (19.3 mg, 0.07 mmol) in 1,4-dioxane (0.8 mL) and the whole mixture was stirred for 18 h. H_2_O was added to the mixture and the whole mixture was extracted with CHCl_3_. The organic layer was dried over Na_2_SO_4_ and was concentrated in vacuo. The crude material was purified by column chromatography (SiO_2_; CHCl_3_/MeOH/H_2_O = 100:3:1, lower phase) to afford **7** (14.3 mg, 54%).

[α]^24^_D_ +15.5° (*c* = 1.24 in CHCl_3_). ^1^H-NMR (500 MHz, CDCl_3_) δ: 9.22 (s, 1H), 8.49 (s, 1H), 7.78 (s, 1H), 7.75 (d, *J* = 8.6 Hz, 1H), 7.62 (d, *J* = 5.7 Hz, 1H), 7.55 (d, *J* = 8.6 Hz, 1H), 6.16 (s, 1H), 5.76 (s, 1H), 5.00 (d, *J* = 10.9 Hz, 1H), 3.03 (t, *J* = 9.5 Hz, 1H), 2.44–2.34 (m, 3H), 2.20 (s, 3H), 2.17–2.13 (m, 3H), 1.76–1.72 (m, 1H), 1.61 (dd, *J* = 12.1, 3.5 Hz, 1H), 1.51 (td, *J* = 12.6, 6.3 Hz, 1H), 0.62 (s, 3H). ^13^C-NMR (125 MHz, CDCl_3_) δ: 163.8, 162.4, 161.6, 152.3, 142.6, 139.0, 134.7, 132.1, 131.4, 128.2, 126.3, 125.9, 120.1, 111.2, 99.7, 98.1, 80.8, 56.6, 55.4, 46.7, 36.8, 29.5, 26.3, 23.8, 20.1, 12.4. ESI MS: *m*/*z* 400 (M + H)^+^. HR-ESI MS: *m*/*z* 400.1907, calcd for C_26_H_26_NO_3_. Found: 400.1902.

#### 3.6.2. (3*S*,3a*R*,11a*S*,11b*R*)-10-(Hydroxymethyl)-3-(isoquinolin-7-yl)-3a,9-dimethyl-1,2,3,3a,4,5,11a,11b-octahydro-7*H*-cyclopenta[h]pyrano[4,3-b]chromen-7-one (**11**)

Compound **10** (27.3 mg, 0.18 mmol) and piperidine (8.7 µL, 0.09 mmol) were added to a solution of **4** (12.9 mg, 0.04 mmol) in 1,4-dioxane (0.8 mL) and the whole mixture was stirred for 22 h. H_2_O was added to the mixture and the whole mixture was extracted with CHCl_3_. The organic layer was dried over Na_2_SO_4_ and was concentrated in vacuo. The crude material was purified by column chromatography (SiO_2_; CHCl_3_/MeOH/H_2_O = 100:3:1, lower phase) to afford **11** (14.3 mg, 76%).

[α]^22^_D_ +22.4° (*c* = 0.93 in CHCl_3_). ^1^H-NMR (500 MHz, CDCl_3_) δ: 9.22 (s, 1H), 8.48 (d, *J* = 5.7 Hz, 1H), 7.78–7.76 (m, 2H), 7.64 (d, *J* = 5.7 Hz, 1H), 7.55 (d, *J* = 8.6 Hz, 1H), 6.16 (s, 1H), 5.05 (d, *J* = 10.9 Hz, 1H), 4.46 (s, 2H), 3.02 (t, *J* = 9.5 Hz, 1H), 2.46–2.34 (m, 3H), 2.32 (s, 3H) 2.23–2.12 (m, 4H), 1.81–1.76 (m, 1H), 1.62 (dd, *J* = 12.1, 5.2 Hz, 1H), 1.51 (td, *J* = 12.1, 5.2 Hz, 1H), 0.62 (s, 3H). ^13^C-NMR (125 MHz, CDCl_3_) δ: 162.5, 161.6, 160.7, 152.2, 142.4, 138.9, 134.8, 132.1, 131.4, 128.5, 126.3, 125.9, 120.2, 111.2, 110.4, 98.7, 81.2, 56.6, 55.5, 55.4, 46.8, 36.8, 29.4, 26.2, 23.8, 17.3, 12.3. ESI MS: *m*/*z* 430 (M + H)^+^. HR-ESI MS: *m*/*z* 430.2013, calcd for C_27_H_28_NO_4_. Found: 430.2025.

#### 3.6.3. (3*S*,3a*R*,11a*S*,11b*R*)-3-(Isoquinolin-7-yl)-3a-methyl-2,3,3a,4,5,8,11a,11b-octahydrocyclopenta[7,8]chromeno[3,2-c]pyridin-7(1*H*)-one (**13**)

Pyridine-2,4-diol (**12**, 7.9 mg, 0.70 mmol) and ethylenediamine diacetate (1.3 mg, 0.007 mmol) were added to a solution of **4** (10.3 mg, 0.035 mmol) in DMF (0.35 mL) at 0 °C, and the whole mixture was stirred at 50 °C for 5 h. H_2_O was added to the mixture and the whole mixture was extracted with CHCl_3_. The organic layer was dried over Na_2_SO_4_ and was concentrated in vacuo. The crude material was purified by column chromatography (SiO_2_; CHCl_3_/MeOH/H_2_O = 60:3:1, lower phase) to afford **13** (3.7 mg, 27%).

[α]^20^_D_ +9.5° (*c* = 0.077 in CHCl_3_). ^1^H NMR (500 MHz, CDCl_3_) δ: 11.05 (brs, 1H), 9.22 (s, 1H), 8.48 (d, *J* = 5.6 Hz, 1H), 7.79 (s, 1H), 7.75 (d, *J* = 8.3 Hz, 1H), 7.62 (d, *J* = 6.0 Hz, 1H), 7.56 (d, *J* = 8.5 Hz, 1H), 7.06 (d, *J* = 7.5 Hz, 1H), 6.36 (s, 1H), 5.89 (d, *J* = 7.2 Hz, 1H), 4.98 (d, *J* = 10.3 Hz, 1H), 3.03 (t, *J* = 9.5 Hz, 1H), 2.47 (dd, *J* = 15.8, 4.3 Hz, 1H), 2.41–2.34 (m, 2H), 2.22–2.15 (m, 3H), 1.80–1.72 (m, 1H), 1.63–1.49 (m, 2H), 0.62 (s, 3H). ^13^C NMR (151 MHz, CDCl_3_) δ: 162.7, 162.46, 152.3, 142.6, 139.2, 134.7, 133.2, 132.3, 132.2, 132.0, 126.3, 125.8, 120.1, 111.9, 100.4, 79.9, 56.7, 55.4, 46.6, 36.7, 29.7, 29.4, 26.4, 24.0, 12.4. ESI MS: *m*/*z* 385 (M + H)^+^. HR-ESI MS: *m*/*z* 385.1911, calcd for C_25_H_25_N_2_O_2_. Found: 385.1935.

#### 3.6.4. (3*S*,3a*R*,11a*S*,11b*R*)-3-(Isoquinolin-7-yl)-3a,8-dimethyl-2,3,3a,4,5,8,11a,11b-octahydrocyclopenta[7,8]chromeno[3,2-c]pyridin-7(1*H*)-one (**15**)

Compound **14** (30 mg, 0.24 mmol) and ethylenediamine diacetate (3.6 mg, 0.02 mmol) were added to a solution of **4** (36.0 mg, 0.12 mmol) in DMF (1.2 mL) at 0 °C, and the whole mixture was stirred at 50 °C for 5 h. H_2_O was added to the mixture and the whole mixture was extracted with CHCl_3_. The organic layer was dried over Na_2_SO_4_ and was concentrated in vacuo. The crude material was purified by column chromatography (SiO_2_; CHCl_3_/MeOH/H_2_O = 60:3:1, lower phase) to afford **15** (28 mg, 57%).

[α]^20^_D_ +21.5° (*c* = 0.271 in CHCl_3_). ^1^H NMR (500 MHz, CDCl_3_) δ 9.23 (s, 1H), 8.49 (s, 1H), 7.79 (s, 1H), 7.75 (d, *J* = 8.5 Hz, 1H), 7.63 (s, 1H), 7.56 (dd, *J* = 8.6, 1.9 Hz, 1H), 7.02 (d, *J* = 7.5 Hz, 1H), 6.42 (s, 1H), 5.82 (d, *J* = 7.5 Hz, 1H), 4.93 (d, *J* = 10.2 Hz, 1H), 3.47 (s, 3H), 3.03 (t, *J* = 8.8 Hz, 1H), 2.47 (dd, *J* = 15.2, 3.3 Hz, 1H), 2.40–2.32 (m, 2H), 2.22–2.14 (m, 3H), 1.78–1.72 (m, 1H), 1.62–1.58 (m, 1H), 1.51 (td, *J* = 13.1, 5.2 Hz, 1H), 0.61 (s, 3H). ^13^C NMR (125 MHz, CDCl_3_) δ: 160.85, 160.82, 152.5, 142.7, 139.4, 136.8, 136.7, 134.8, 132.3, 132.0, 126.4, 125.9, 120.3, 112.9, 107.5, 99.6, 79.8, 56.8, 55.4, 46.7, 37.3, 36.8, 29.5, 26.5, 24.2, 12.5. ESI MS: *m*/*z* 399 (M + H)^+^. HR-ESI MS: *m*/*z* 399.2067, calcd for C_26_H_27_N_2_O_2_. Found: 399.2085.

#### 3.6.5. 4-Hydroxy-5-(hydroxymethyl)-1-methylpyridin-2(1*H*)-one (**18**)

Diisobutylaluminium hydride (1.0 M in toluene, 1.2 mL, 1.2 mmol) was added to a solution of **17** (50 mg, 0.30 mmol) in toluene (2.4 mL) at –78 °C and the whole mixture was stirred at 0 °C for 7 h. Then, 5% HCl aq. was added to quench the reaction, and the whole mixture was concentrated in vacuo. The crude material was purified by column chromatography (SiO_2_; CHCl_3_/MeOH/H_2_O = 6:4:1) to afford **18** (16.6 mg, 39%).

^1^H NMR (500 MHz, DMSO-*d*_6_) δ: 10.63 (s, 1H), 7.43 (s, 1H), 5.62 (s, 1H), 4.88 (s, 1H), 4.22 (s, 2H), 3.37 (s, 3H). ^13^C NMR (125 MHz, DMSO-*d*_6_) δ: 165.0, 163.0, 137.7, 112.4, 97.7, 56.1, 35.8. ESI MS: *m*/*z* 156 (M + H)^+^. HR-ESI MS: *m*/*z* 156.0655, calcd for C_7_H_9_NO_3_. Found: 156.0660.

#### 3.6.6. (3*S*,3a*R*,11a*S*,11b*R*)-10-(Hydroxymethyl)-3-(isoquinolin-7-yl)-3a,8-dimethyl-2,3,3a,4,5,8,11a,11b-octahydrocyclopenta[7,8]chromeno[3,2-c]pyridin-7(1*H*)-one (**19**)

Compound **18** (18.6 mg, 0.12 mmol) and ethylenediamine diacetate (2.2 mg, 0.01 mmol) were added to a solution of **4** (18.0 mg, 0.062 mmol) in DMF (0.6 mL) at 0 °C and the whole mixture was stirred at 50 °C for 5 h. H_2_O was added to the mixture and the whole mixture was extracted with CHCl_3_. The organic layer was dried over Na_2_SO_4_ and was concentrated in vacuo. The crude material was purified by column chromatography (SiO_2_; CHCl_3_/MeOH/H_2_O = 60:3:1, lower phase) to afford **19** (11.8 mg, 45%).

[α]^20^_D_ +14.2° (*c* = 0.361 in CHCl_3_). ^1^H NMR (600 MHz, CDCl_3_) δ: 9.21 (s, 1H), 8.48 (s, 1H), 7.78 (s, 1H), 7.75 (d, *J* = 8.5 Hz, 1H), 7.63 (d, *J* = 5.5 Hz, 1H), 7.55 (d, *J* = 8.3 Hz, 1H), 7.10 (s, 1H), 6.40 (s, 1H), 4.97 (d, *J* = 11.0 Hz, 1H), 4.44 (dd, *J* = 28.9, 12.9 Hz, 2H), 3.46 (s, 3H), 3.01 (t, *J* = 9.3 Hz, 1H), 2.90–2.60 (br, 1H), 2.47 (m, 1H), 2.41–2.31 (m, 2H), 2.23–2.11 (m, 3H), 1.81–1.72 (m, 1H), 1.61 (m, 1H), 1.51 (m, 1H), 0.61 (s, 3H). ^13^C NMR (151 MHz, CDCl_3_) δ: 160.5, 158.8, 152.2, 142.5, 139.1, 135.2, 134.7, 132.2, 132.1, 129.1, 126.3, 125.8, 120.1, 112.6, 111.6, 106.8, 79.9, 58.3, 56.6, 55.4, 46.6, 37.1, 36.8, 29.4, 26.3, 23.9, 12.4. ESI MS: *m*/*z* 429 (M + H)^+^. HR-ESI MS: *m*/*z* 429.2173, calcd for C_27_H_29_N_2_O_3_. Found: 429.2199.

#### 3.6.7. *tert*-Butyl (2-(2,2-Dimethyl-4-oxo-4*H*-1,3-dioxin-6-yl)-3-oxobutyl)carbamate (**22**)

Sodium hydride (335 mg, 8.38 mmol) was added to a solution of **21** (616 mg, 3.35 mmol) in THF (33.5 mL) at 0 °C, and the whole mixture was stirred at rt for 10 min. A solution of *tert*-butyl (tosylmethyl)carbamate (1.4 g, 5.0 mmol) in THF (10 mL) was added to the reaction mixture at 0 °C and the whole mixture was stirred for 1 h. Sat. NH_4_Cl aq. was added to quench the reaction, and the whole mixture was extracted with Et_2_O. The organic layer was dried over Na_2_SO_4_ and was concentrated in vacuo. The crude material was purified by column chromatography (SiO_2_; *n*-hexane/AcOEt = 2:1) to afford **22** (581 mg, 55%).

^1^H NMR (500 MHz, CDCl_3_) δ: 5.36 (s, 1H), 4.96 (t, *J* = 6.4 Hz, 1H), 3.63 (t, *J* = 6.8 Hz, 1H), 3.52–3.47 (m, 1H), 3.42–3.37 (m, 1H), 2.20 (s, 3H), 1.64 (s, 6H), 1.46 (s, 9H). ^13^C NMR (125 MHz, CDCl_3_) δ: 202.6, 165.4, 160.1, 155.6, 107.1, 96.4, 79.8, 57.0, 38.7, 29.2, 28.2, 24.8, 24.7. ESI MS: *m*/*z* 336 (M + Na)^+^. HR-ESI MS: *m*/*z* 336.1418, calcd for C_15_H_23_NO_6_Na. Found: 336.1434.

#### 3.6.8. *tert*-Butyl (*Z*)-(5-(methylamino)-2-(1-(methylamino)ethylidene)-3,5-dioxopentyl)carbamate (**23**)

Methylamine (2.0 M in THF, 0.09 mL, 0.18 mmol) was added to a solution of **22** (28.4 mg, 0.09 mmol) in toluene (4.5 mL) and the whole mixture was stirred at 75 °C for 4 h. Concentration in vacuo gave the crude material, which was purified by column chromatography (SiO_2_; CHCl_3_/MeOH/H_2_O = 200:3:1, lower phase) to afford **23** (13.4 mg, 50%).

^1^H NMR (500 MHz, CDCl_3_) δ: 12.24 (brs, 1H), 6.94 (brs, 1H), 5.32 (brs, 1H), 3.99 (d, *J* = 5.0 Hz, 2H), 3.34 (s, 2H), 2.94 (d, *J* = 5.0 Hz, 3H), 2.76 (d, *J* = 5.0 Hz, 3H), 2.08 (s, 3H), 1.39 (s, 9H). ^13^C NMR (125 MHz, CDCl_3_) δ: 189.4, 168.9, 168.8, 155.6, 101.7, 78.9, 46.4, 39.4, 30.1, 28.3, 26.3, 15.1. ESI MS: *m*/*z* 322 (M + Na)^+^. HR-ESI MS: *m*/*z* 322.1737, calcd for C_14_H_25_N_3_O_4_Na. Found: 322.1749.

#### 3.6.9. *N*-((4-Hydroxy-1,2-dimethyl-6-oxo-1,6-dihydropyridin-3-yl)methyl)acetamide (**26**)

AcOH (14 µL, 0.48 mmol) was added to a solution of **23** (72 mg, 0.24 mmol) in toluene (2.4 mL) and the whole mixture was stirred at 110 °C for 8 h. Concentration in vacuo gave the crude material including compound **24**, which was dissolved in CH_2_Cl_2_ (1.2 mL). TFA (0.24 mL) was added to the solution and the whole mixture was stirred for 1 h. The reaction mixture was diluted with toluene (10 mL) and the whole mixture was concentrated in vacuo. The residue was dissolved in DMF (1.2 mL), and then K_2_CO_3_ (138 mg, 1.2 mmol) and Ac_2_O (28 µL, 0.30 mmol) were successively added to the solution. The whole mixture was stirred for 2 h. MeOH (1.0 mL) was added to the reaction mixture, and the whole mixture was stirred for additional 2h. Concentration in vacuo gave the crude material, which was purified by column chromatography (SiO_2_; CHCl_3_/MeOH/H_2_O = 30:3:1, lower phase) to afford **26** (17.0 mg, 34% in 3 steps).

^1^H NMR (500 MHz, CD_3_OD/CDCl_3_) δ: 5.81 (s, 1H), 4.25 (s, 2H), 3.49 (s, 3H), 2.43 (s, 3H), 1.91 (s, 3H). ^13^C NMR (125 MHz, CD_3_OD/CDCl_3_) δ: 172.7, 167.1, 166.3, 148.3, 110.3, 97.3, 35.5, 31.8, 22.4, 17.0. ESI MS: *m*/*z* 233 (M + Na)^+^. HR-ESI MS: *m*/*z* 233.0897, calcd for C_10_H_14_N_2_O_3_. Found: 233.0908.

#### 3.6.10. *N*-(((3*S*,3a*R*,11a*S*,11b*R*)-3-(Isoquinolin-7-yl)-3a,8,9-trimethyl-7-oxo-1,2,3,3a,4,5,7,8,11a,11b-decahydrocyclopenta[7,8]chromeno[3,2-c]pyridin-10-yl)methyl)acetamide (**27**)

Compound **26** (15.3 mg, 0.068 mmol) and ethylenediamine diacetate (1.3 mg, 0.007 mmol) were added to a solution of **4** (10.0 mg, 0.034 mmol) in DMF (0.34 mL) at 0 °C and the whole mixture was stirred at 50 °C for 5 h. H_2_O was added to the mixture and the whole mixture was extracted with CHCl_3_. The organic layer was dried over Na_2_SO_4_ and was concentrated in vacuo. The crude material was purified by column chromatography (SiO_2_; CHCl_3_/MeOH/H_2_O = 100:3:1, lower phase) to afford **27** (5.0 mg, 30%).

[α]^20^_D_ −8.5° (*c* = 0.138 in CHCl_3_). ^1^H NMR (500 MHz, CDCl_3_) δ: 9.25 (brs, 1H), 8.50 (brs, 1H), 7.81 (s, 1H), 7.78 (d, *J* = 8.5 Hz, 1H), 7.66 (brs, 1H), 7.58 (d, *J* = 8.6 Hz, 1H), 6.41 (s, 1H), 5.90 (brs, 1H), 4.95 (d, *J* = 10.8 Hz, 1H), 4.32 (dd, *J* = 14.1, 5.5 Hz, 1H), 4.23 (dd, *J* = 14.4, 5.2 Hz, 1H), 3.50 (s, 3H), 3.04 (t, *J* = 10.1 Hz, 1H), 2.49 (dd, *J* = 15.2, 5.2 Hz, 1H), 2.43–2.33 (m, 2H), 2.41 (s, 3H), 2.23–2.13 (m, 3H), 2.00 (s, 3H), 1.83–1.76 (m, 1H), 1.63 (dd, *J* = 12.6, 4.7 Hz, 1H), 1.52 (td, *J* = 12.8, 5.6 Hz, 1H), 0.63 (s, 3H). ^13^C NMR (151 MHz, CDCl_3_) δ 169.8, 160.5, 158.8, 152.3, 144.9, 142.6, 138.9, 134.7, 132.0, 131.3, 126.3, 125.8, 120.1, 113.0, 111.6, 107.0, 104.3, 80.0, 56.6, 55.40, 46.6, 36.8, 34.4, 31.5, 29.2, 26.3, 24.1, 23.2, 17.0, 12.3. ESI MS: *m*/*z* 484 (M + H)^+^. HR-ESI MS: *m*/*z* 484.2595, calcd for C_30_H_34_N_3_O_3_. Found: 484.2623.

## 4. Conclusions

In summary, we developed a synthetic method for novel simplified analogs of cortistatin A (**1**) using 4-hydroxy-2-pyrone or 4-hydroxy-2-pyridone derivatives as A-ring fragments. Among those prepared in this work, the pyridone-embedded analog **19** is the most potent compound exhibiting significant in vivo anti-angiogenic and anti-tumor effect without acute toxicity and hepatotoxicity. Although some improvements in the reaction yield for the synthesis are needed, easy accessibility and more potent activity/selectivity than those of natural products are significant features of this compound, making it a promising candidate for anti-tumor drugs. Further studies to develop a more practical compound and elucidate its mechanism of action in angiogenesis are currently underway.

## Figures and Tables

**Table 1:** Antiproliferative activities of cortistatin A analogs.

Compound	IC_50_ (µM)		S.I. ^a^
	HUVECs	KB3-1	
**1**	0.0018 ^b^	7.0 ^b^	3900 ^b^
**2**	0.035 ^c^	10.5 ^c^	300 ^c^
**3**	0.0026 ^c^	8.2 ^c^	3150 ^c^
**7**	0.09	62.8	700
**11**	0.02	10	500
**13**	0.01	11	1100
**15**	0.003	8.6	2870
**19**	0.001	6.4	6400
**27**	0.01	13.4	1340
**28**	0.56	4.8	8.6

^a^ S.I. = selective index: IC_50_ against KB3-1 cells/IC_50_ against HUVECs. ^b^ Reported IC_50_ values in ref. [8]. ^c^ Reported IC_50_ values in Ref. [21].

**Table 2:** In vivo anti-angiogenic effect, body weight measurement, and hepatotoxicity evaluation of analog **19**.

		19	Body Weight (g) ^a^		Hb Content	AST	ALT
	bFGF	(mg/kg)	Day 1	Day 9	(µg/mg) ^b^	(IU/L)	(IU/L)
1	–	–	26.03 ± 0.30	26.33 ± 0.33	0.65 ± 0.10	–	–
2	+	–	25.62 ± 0.52	25.98 ± 0.48	8.12 ± 1.25	59.6 ± 3.1	43.6 ± 4.7
3	+	0.1	25.70 ± 0.58	26.12 ± 0.58	3.27 ± 0.83	–	–
4	+	0.3	25.60 ± 0.64	26.14 ± 0.62	2.42 ± 0.79	–	–
5	+	1.0	26.30 ± 0.41	26.64 ± 0.39	0.79 ± 0.24	57.8 ± 4.5	48.4 ± 3.3

^a^ The mean body weight of five mice in each group (mean ± SD). ^b^ Hemoglobin content (µg)/isolated Matrigel (mg) (mean ± SD).

## Data Availability

The original data presented in the study are included in the article/Supplementary Material; further inquiries can be directed to the corresponding author.

## Supplementary Materials

The following supporting information can be downloaded at: https://www.mdpi.com/article/10.3390/md23040179/s1, Supplementary Data S1: NMR spectra of the new compounds.
