# Supplementary material for: Synthesis and Evaluation of Antitumor and Anti-Angiogenesis Activity of Pyrone- or Pyridone-Embedded Analogs of Cortistatin A
Source: Mar Drugs. 2025 Apr 20;23(4):179. doi: 10.3390/md23040179 (PMC12029069; doi:10.3390/md23040179)
Supplement: Supplementary file 1 [file marinedrugs-23-00179-s001.zip › marinedrugs-3588982-supplementary.pdf]

Supporting Information

Synthesis and Biological Evaluation of Pyrone- or Pyridone-Embedded Analogs of Cortistatin A

Yuri Fujimoto, Kanako Mizuno, Yuta Nakamura, Masayoshi Arai, and Naoyuki Kotoku*

College of Pharmaceutical Sciences, Ritsumeikan University, 1-1-1 Noji-higashi, Kusatsu, Shiga, 525-8577, Japan
Graduate School of Pharmaceutical Sciences, Osaka University, 1-6 Yamadaoka, Suita,
Osaka 565-0871, Japan

Contents

^{1}H NMR and ^{13}C NMR spectra for new compounds: pages S2-S11

^{1}H NMR (500 MHz, $CDCl_3$)

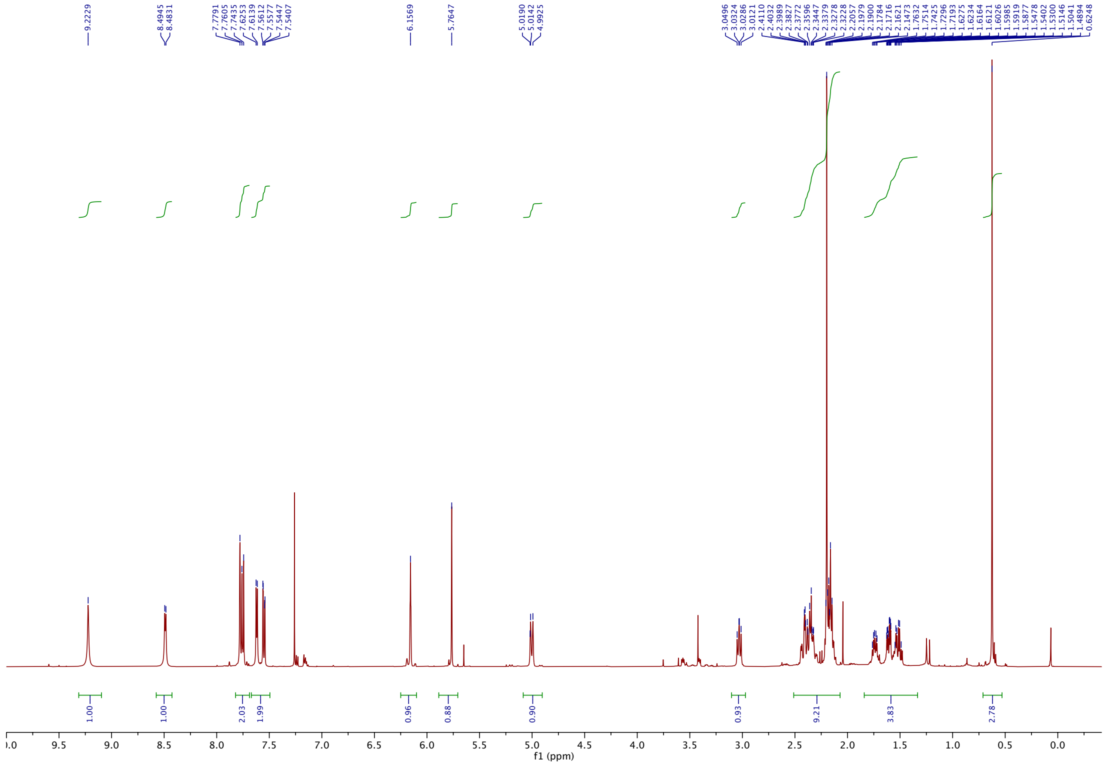

^{13}C NMR (125 MHz, $CDCl_3$)

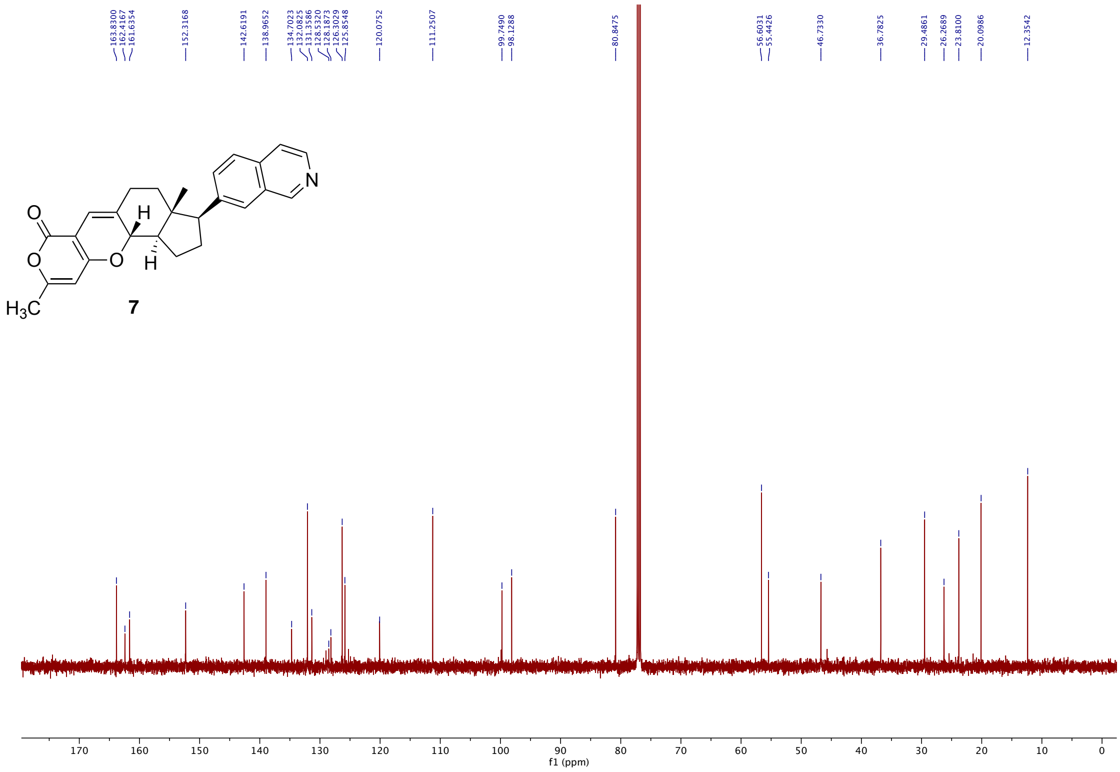

7

^{1}H NMR (500 MHz, $CDCl_3$)

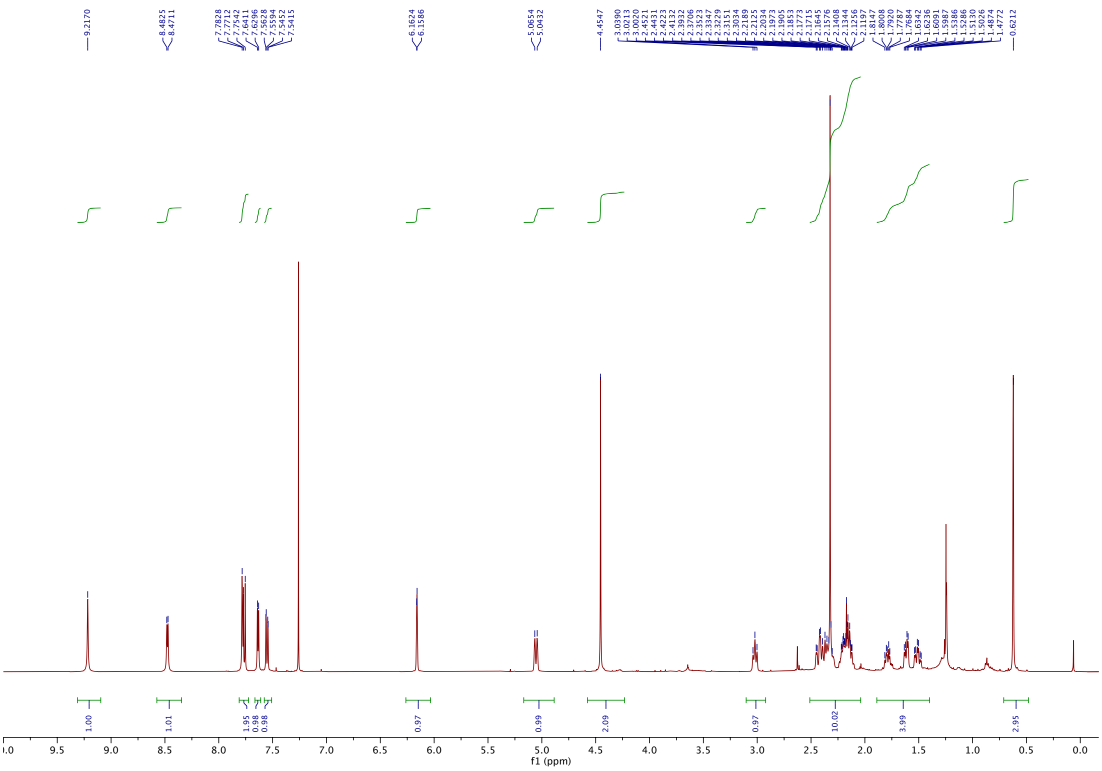

^{13}C NMR (125 MHz, $CDCl_3$)

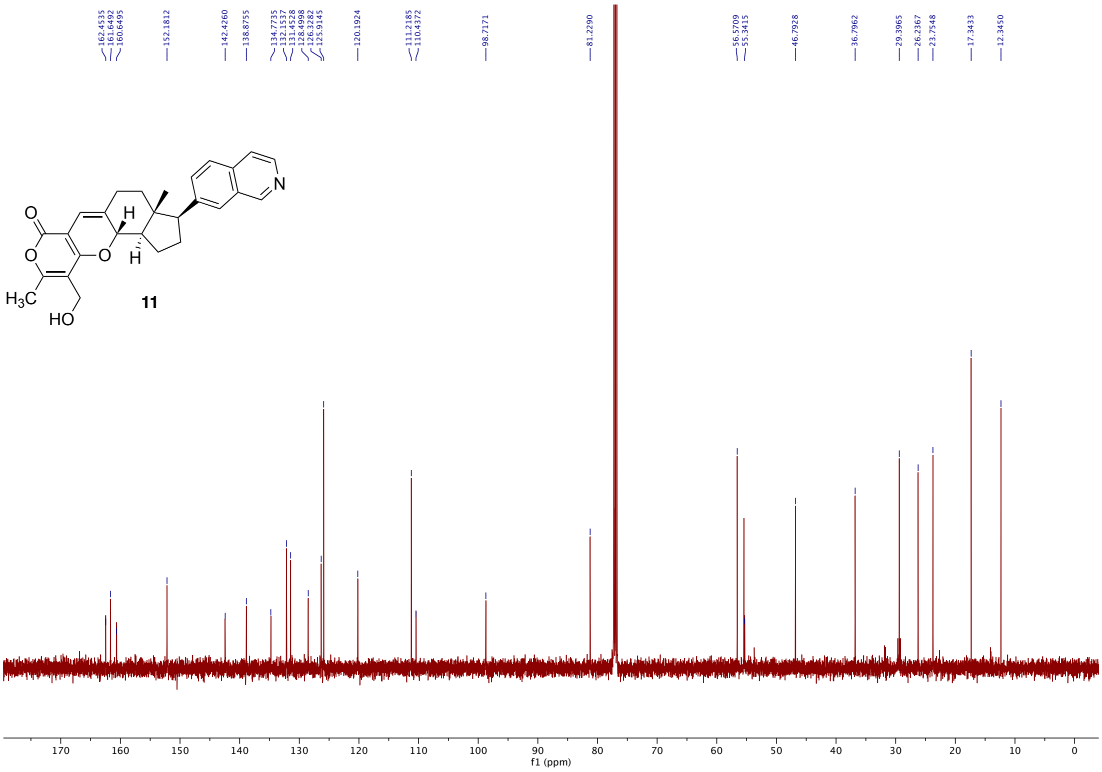

^{1}H NMR (500 MHz, CDCl$_3$)

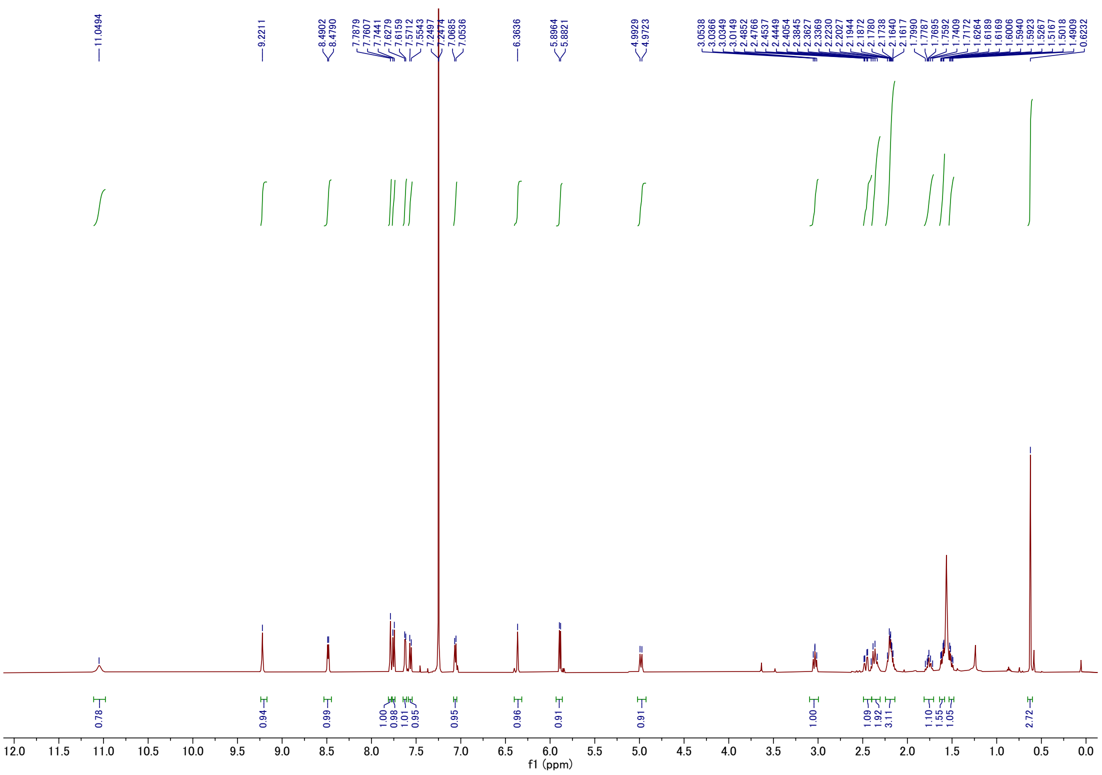

^{13}C NMR (125 MHz, CDCl$_3$)

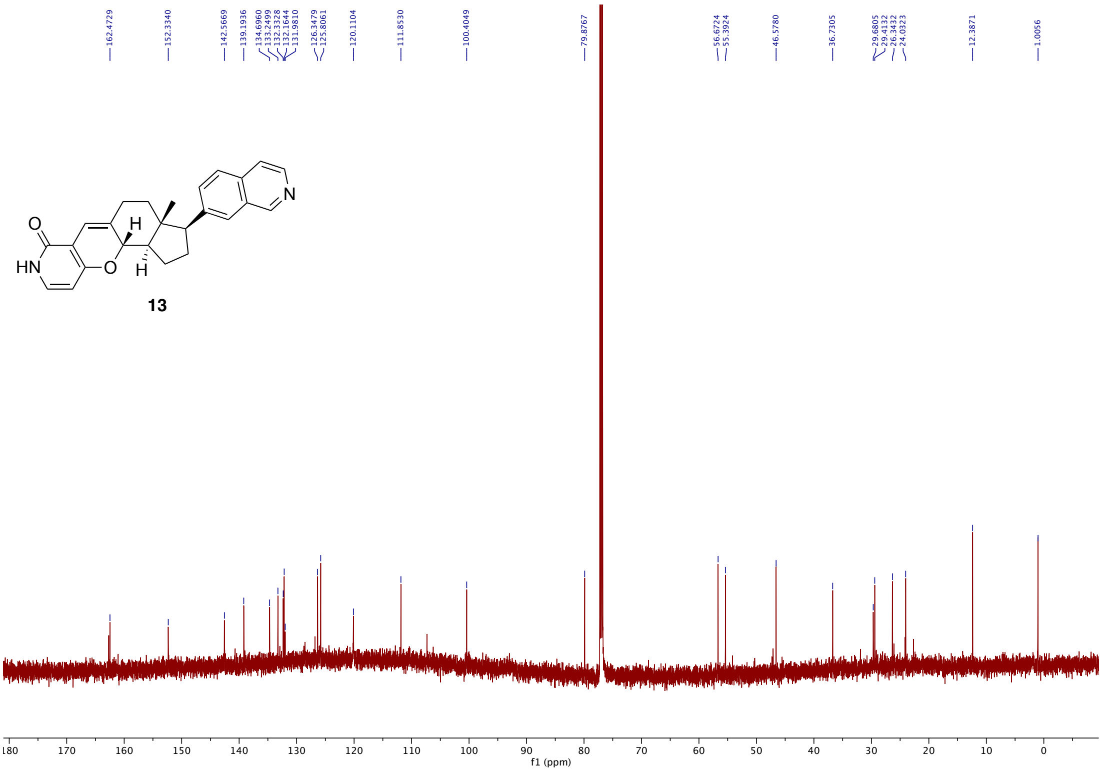

13

^{1}H NMR (500 MHz, $CDCl_3$)

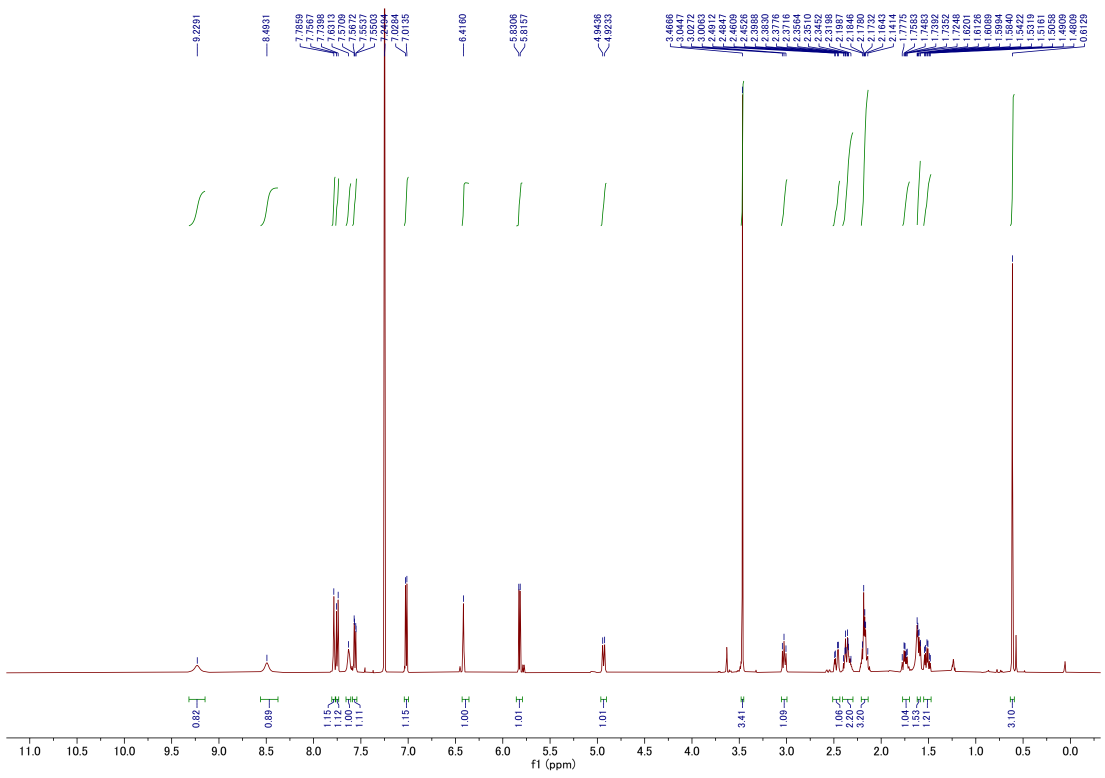

^{13}C NMR (125 MHz, $CDCl_3$)

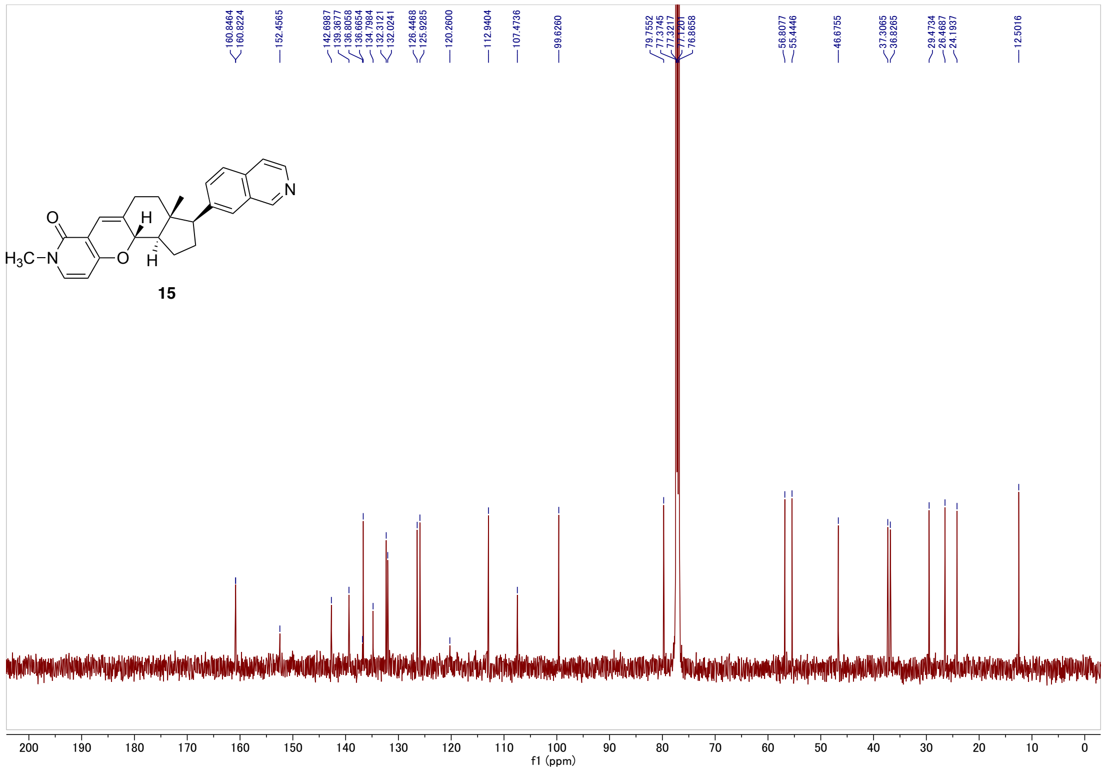

^{1}H NMR (500 MHz, DMSO-d_6)

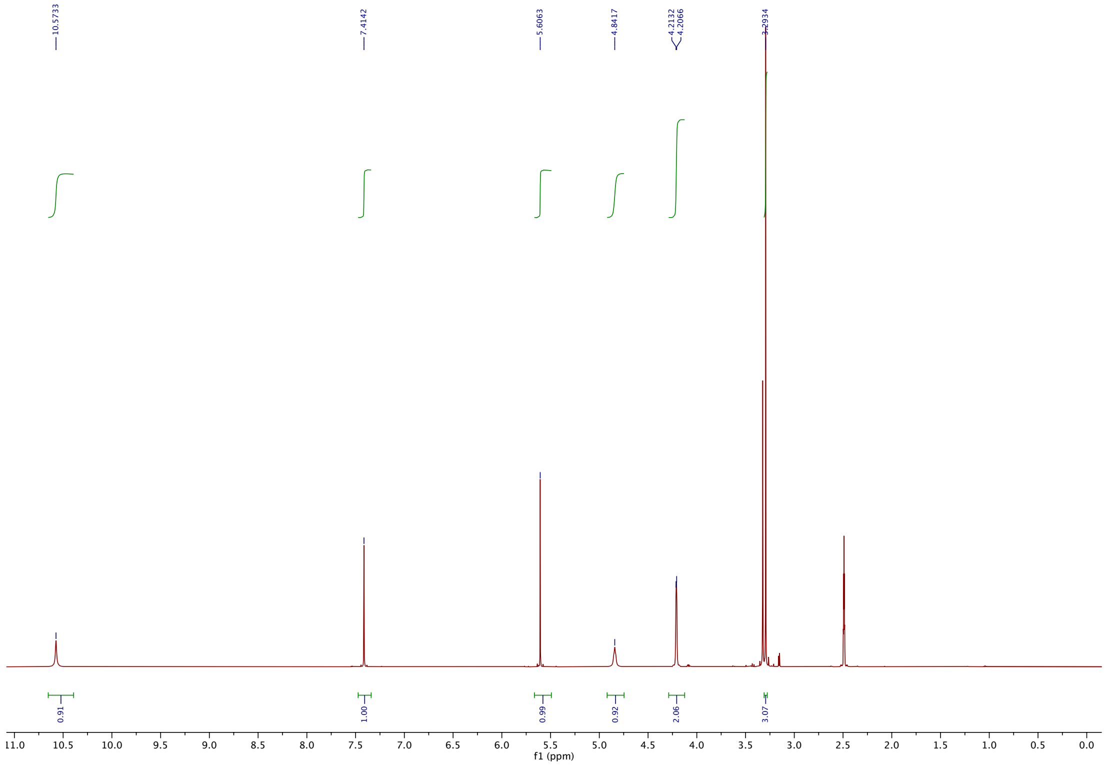

^{13}C NMR (125 MHz, DMSO-d_6)

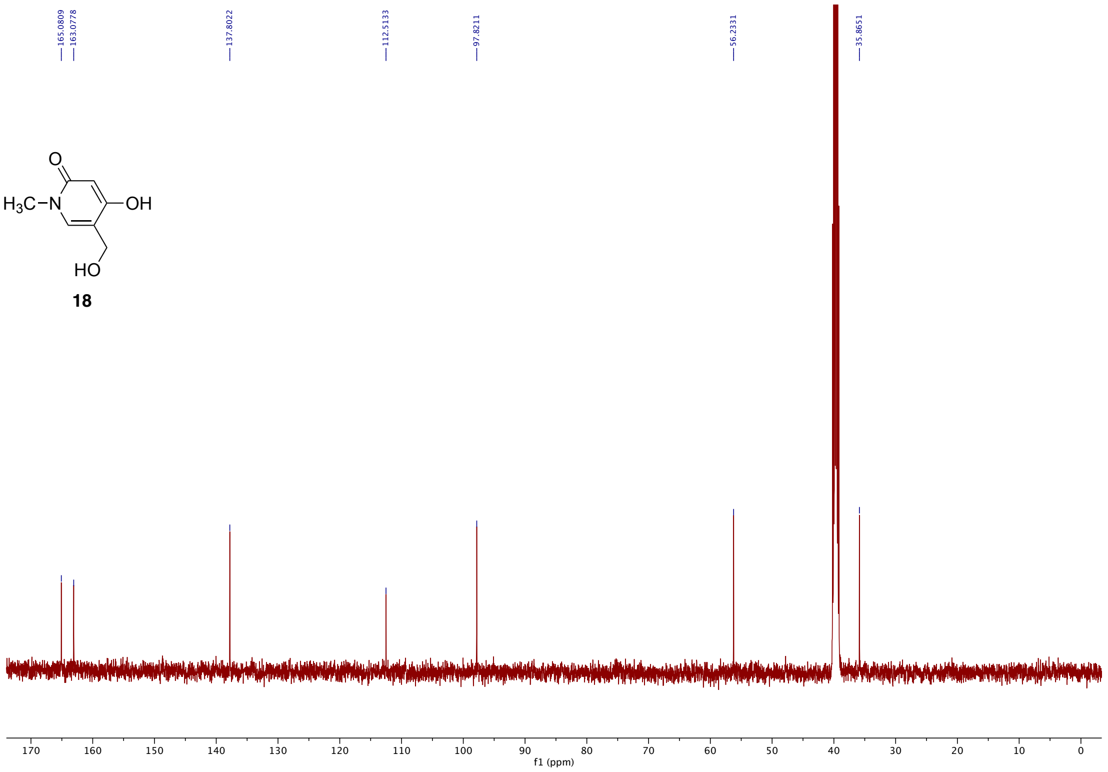

18

^{1}H NMR (500 MHz, $CDCl_3$)

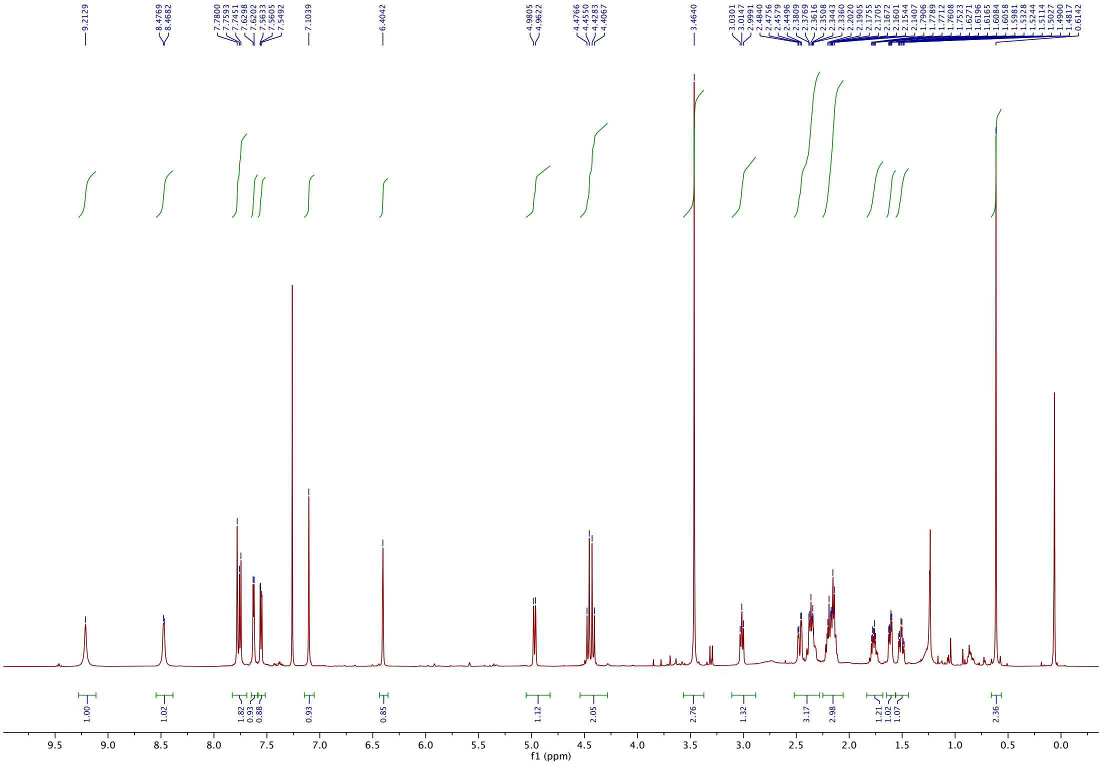

^{13}C NMR (125 MHz, $CDCl_3$)

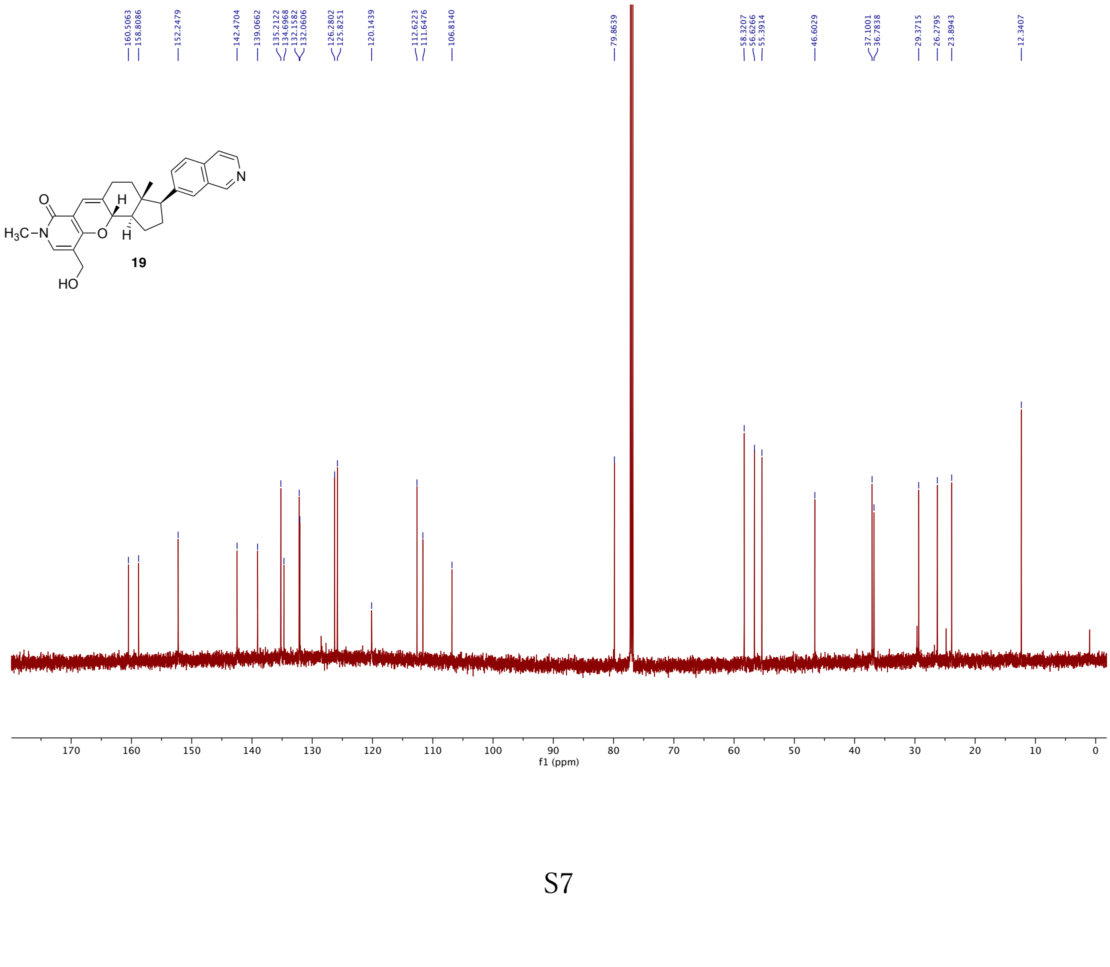

19

^{1}H NMR (500 MHz, $CDCl_3$)

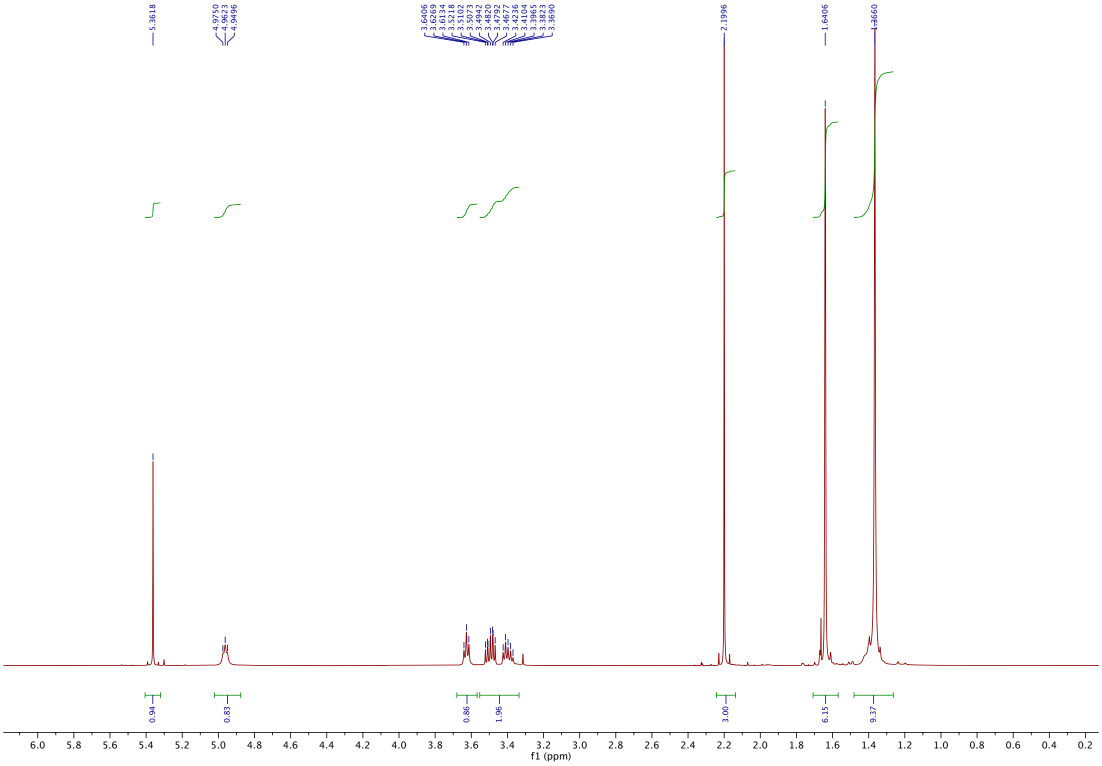

^{13}C NMR (125 MHz, $CDCl_3$)

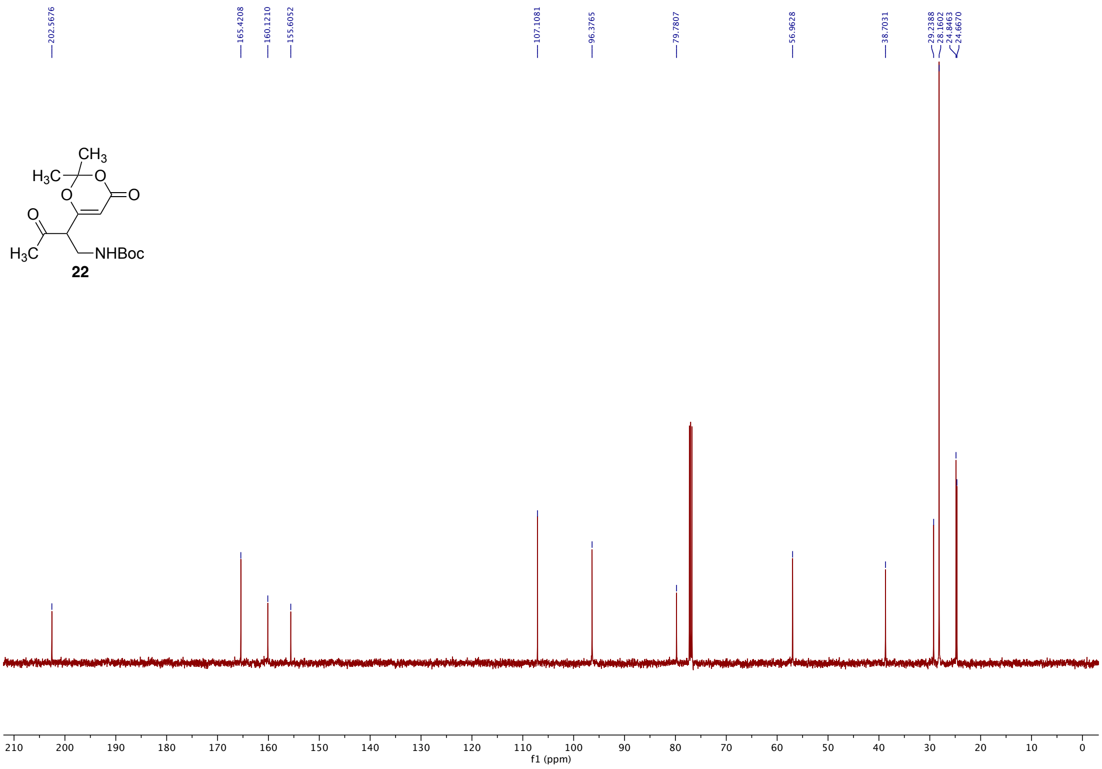

22

^{1}H NMR (500 MHz, $CDCl_3$)

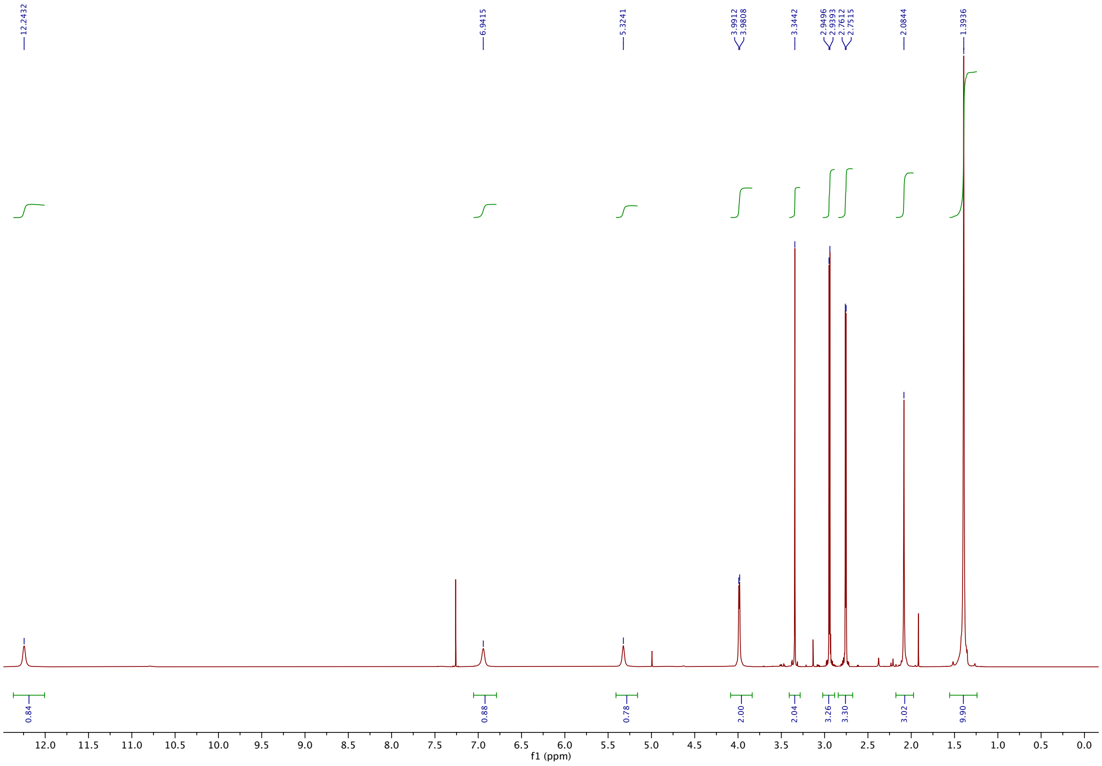

^{13}C NMR (125 MHz, $CDCl_3$)

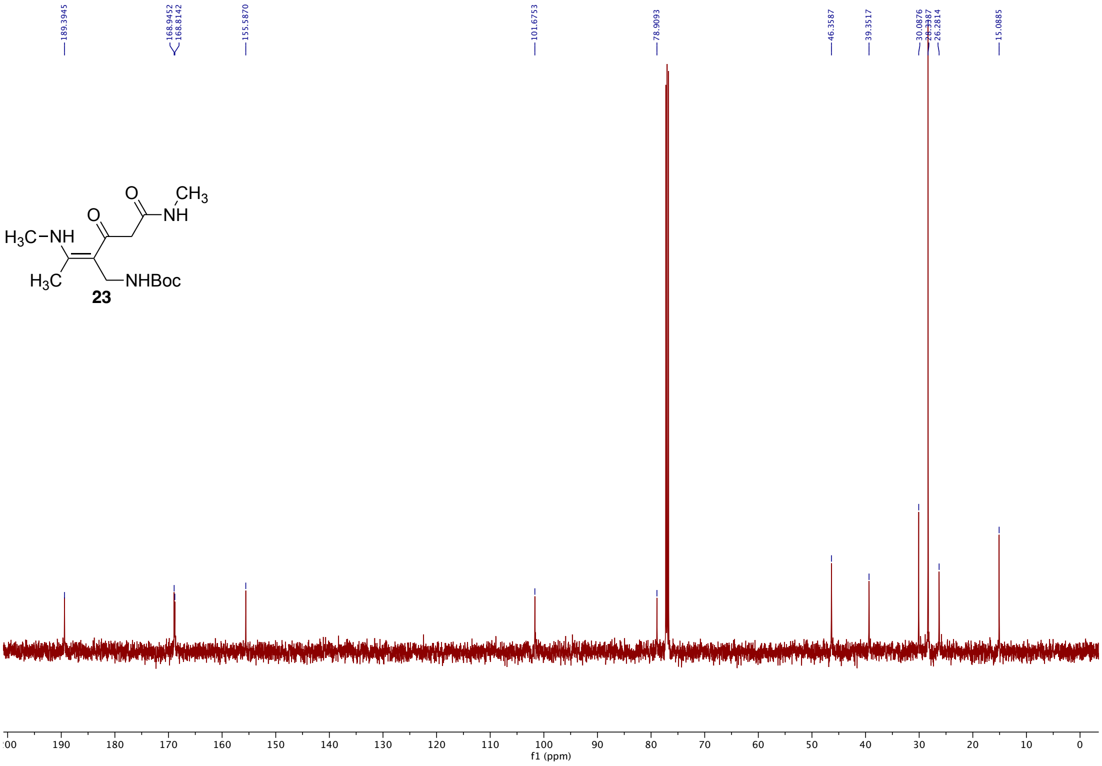

^{1}H NMR (500 MHz, $CD_3OD/CDCl_3$)

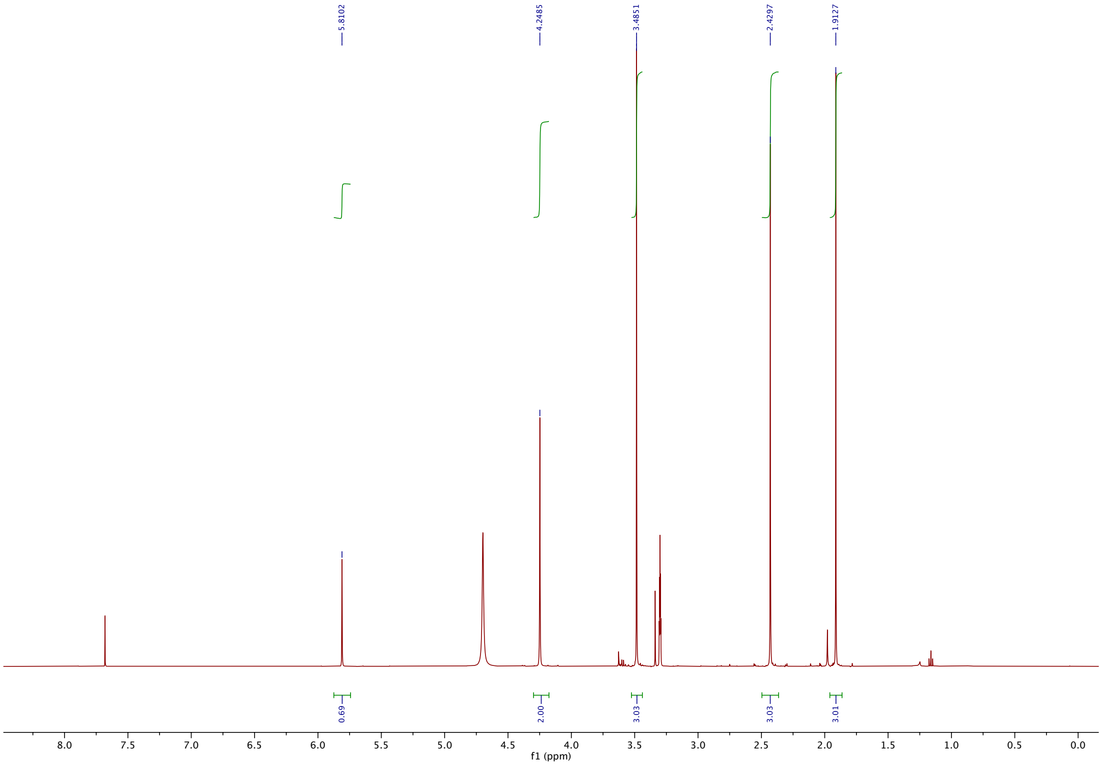

^{13}C NMR (125 MHz, $CD_3OD/CDCl_3$)

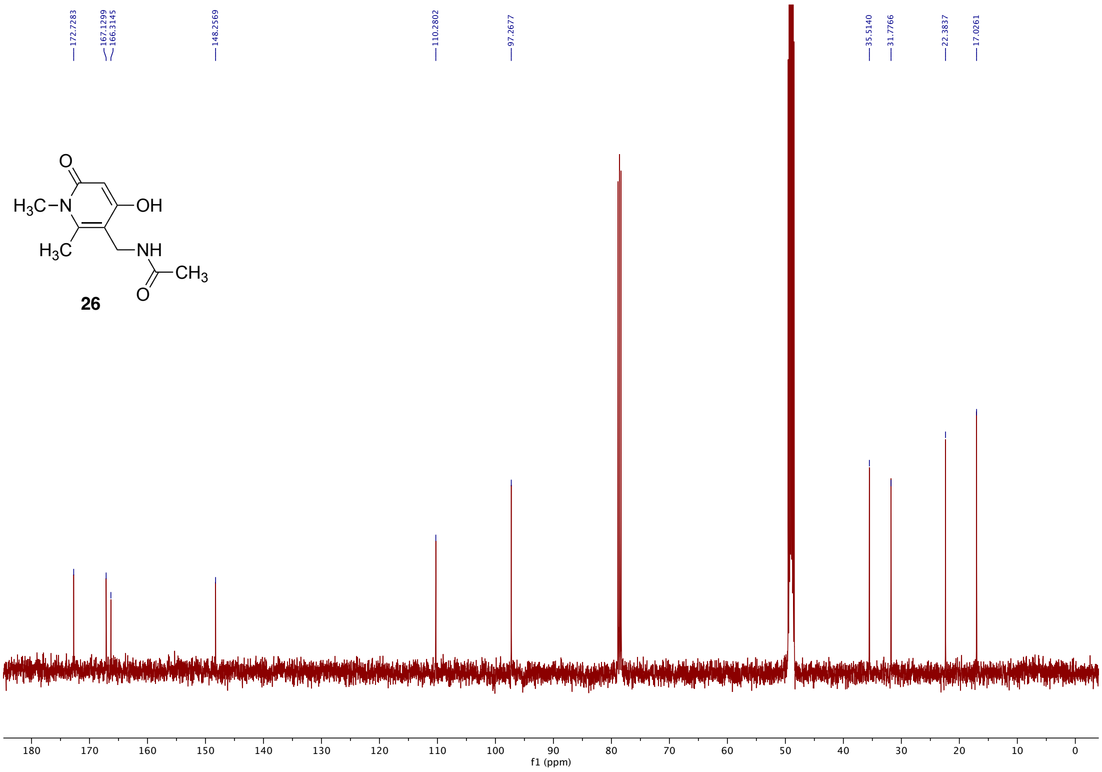

26

^{1}H NMR (500 MHz, $CDCl_3$)

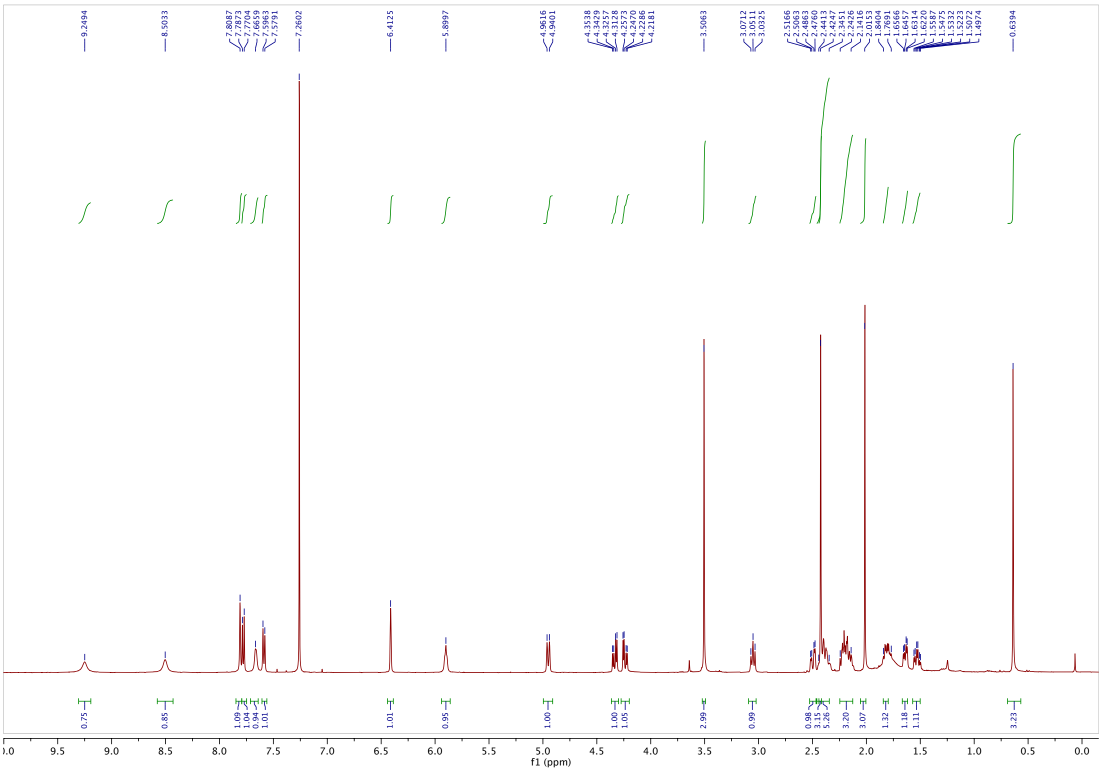

^{13}C NMR (125 MHz, $CDCl_3$)

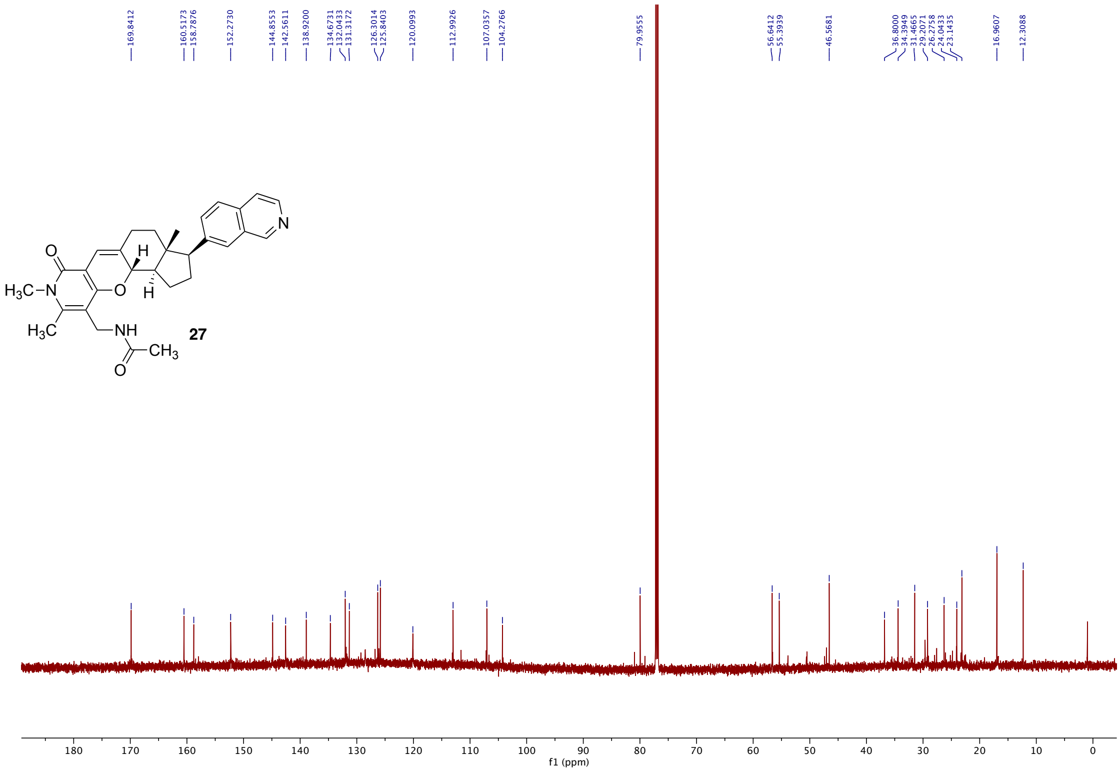

27
